# Recent advances in biomaterials for bone regeneration: Bridging innovation and clinical translation

**DOI:** 10.1016/j.mtbio.2025.102685

**Published:** 2025-12-17

**Authors:** Zahra Sabouri, Mélanie Dequeecker, Houmam Anees, Fatemeh Rastegar Adib, Reem Jamous, Junwen Zheng, Xiaolong Lyu, Sabine Stoetzel, Christian Heiss, Thaqif El Khassawna, Vahid Jahed

**Affiliations:** a Experimental Trauma Surgery, Faculty of Medicine, Justus-Liebig-University, Giessen, Germany; b Department of Trauma, Hand and Reconstructive Surgery, University Hospital of Giessen-Marburg, Giessen, Germany; c Biruni University, Istanbul, 34015, Türkiye; d School of Pharmacy, The University of Jordan, Amman, 11942, Jordan

**Keywords:** Bone regeneration, Mineral-based biomaterials, Natural biomaterials, Synthetic biomaterials, Composite materials

## Abstract

Bone regeneration presents an enduring clinical barrier, particularly with the projected rise in osteoporotic fractures exceeding 6 million annually by 2050. Autografts, allografts, and xenografts remain foundational in bone repair due to their inherent osteogenic, osteoconductive, and osteoinductive capacities. However, issues such as donor site morbidity, immunogenicity, limited graft availability, and pathogen transmission risks limit their applicability. In response, recent developments in biomaterials, including ion-doped bioceramics, bioactive glass-polymer composites, and stem cell-functionalized hydrogels, aim to replicate the hierarchical structure and biochemical microenvironment of native bone. This review surveys advancements in scaffold materials over the past five years, evaluating their physicochemical properties, immune modulation potential, and clinical readiness within the context of bone tissue engineering (BTE). Specific attention is given to strategies for selecting appropriate biomaterials based on clinical needs, considering their physical and biological properties, as well as their respective advantages and limitations. Despite this progress, clinical translation remains limited; only a few engineered scaffolds have achieved regulatory approval for routine use. To accelerate adoption, efforts must focus on scalable fabrication, quantitative immune profiling, and scaffold degradation monitoring to bridge preclinical performance with clinical efficacy.

## Graphical abstract

**Figure:**
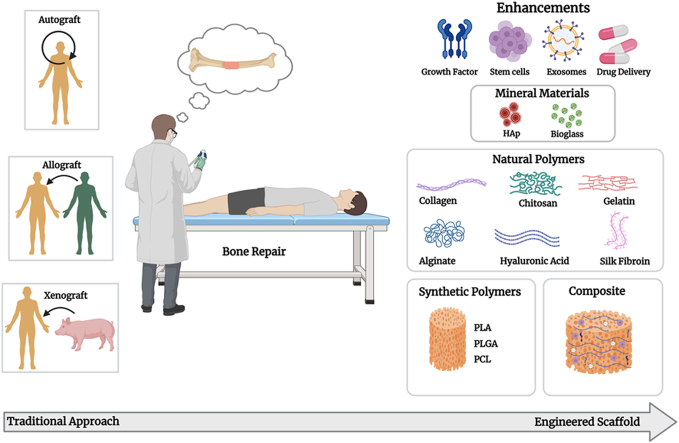

## 1 Introduction

With rising life expectancy, bone health has become a major concern, since the loss of bone mass and structure predisposes individuals to fractures, disability, and reduced quality of life [1]. Bone defects arise from diverse causes, including trauma, infection, tumors, reconstructive procedures, and congenital disorders [2]. Although bone possesses intrinsic regenerative capacity, healing is often slow and limited by factors such as defect size, patient age, vascularization, and mineral availability [[3], [4], [5]]. In cases of critical-size defects, surgical intervention with bone grafts is frequently required [6]. Autografts, allografts, and xenografts have received significant clinical attention; however, limitations such as donor site morbidity, immune rejection, and disease transmission have prompted the search for alternative strategies [4,7,8].

Recent advances in material science have accelerated the development of innovative natural and synthetic biomaterials designed to mimic the composition and architecture of bone [[9], [10], [11], [12]]. These are often combined with cells, growth factors, or nanotechnology-based modifications to enhance osteogenesis and enable controlled biodegradation [[13], [14], [15], [16]]. Despite these advances, the clinical translation of engineered scaffolds remains limited, with only a few products achieving routine use. This review offers a thorough evaluation of the latest progress in biomaterials for bone repair over the past five years, outlining their advantages as well as persistent challenges. It examines existing commercial products to demonstrate how problems like mechanical incompatibility, degradation management, and immune system compatibility are being tackled, while also pinpointing remaining gaps in clinical application. Future outlook focuses on key mechanical and biological factors crucial for scaffold design, such as stiffness, porosity, degradation rate, cell adhesion, mineralization, and macrophage polarization, and connects these to particular defect types and clinical needs. By combining these elements, the review provides a framework for customizing biomaterials to ensure structural integrity, modulate immune responses, and support effective bone healing.

## 2 Traditional treatments for bone defects

Autografts remain the gold standard for bone defect repair, as they provide osteogenic, osteoconductive, and osteoinductive properties [10,[17], [18], [19]]. Clinical studies confirm their strong efficacy; for example, bone marrow–derived mesenchymal stem cells (BM-MSCs) combined with bioceramics achieved consolidation rates of up to 92.8 % in non-unions [20]. However, autografts are constrained by donor site morbidity, pain, infection risk, and high cost. Iliac crest harvesting, the most common source, may damage surrounding tissues, increase surgical complexity, and lead to necrosis [10,18]. To address these limitations, clinicians have turned to allografts, which offer cancellous, cortical, or demineralized bone matrix (DBM) options with osteoconductive and, in some cases, osteoinductive potential [21]. Freeze-dried allografts are FDA-approved and can be osteogenic, but sterilization procedures compromise their mechanical strength and reduce osteoinduction [22]. DBM promotes osteoblast proliferation but lacks sufficient mechanical stability for load-bearing applications [[22], [23], [24], [25], [26], [27], [28]].

**Fig. 1:**
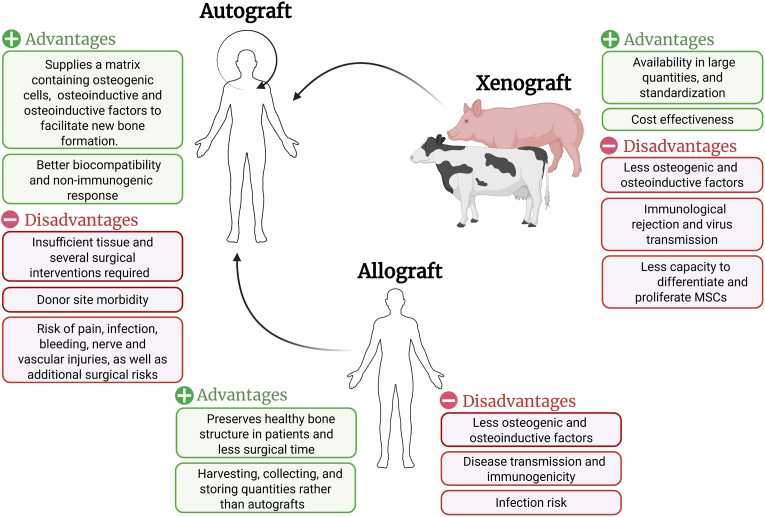
Comparative advantages and disadvantages of autografts, allografts, and xenografts in bone defect repair. Autografts provide optimal osteogenic potential and biocompatibility but are limited by donor site morbidity and supply. Allografts reduce surgical time but carry risks of infection, reduced mechanical strength, and immunogenicity. Xenografts are abundant and cost-effective but demonstrate limited osteoinduction and risk of disease transmission.

As an alternative, xenografts derived from non-human species offer cost-effective and widely available options. Bovine cancellous xenografts demonstrate good osteointegration [[29], [30], [31]], but sterilization reduces osteogenic potential and risks of viral transmission remain [32,33]. Combining xenografts with morphogenetic proteins or autologous bone marrow can enhance outcomes, but these remain adjunct strategies [11,34]. However, all graft types face limitations of supply, immunogenicity, and mechanical performance, prompting biomaterial innovation. These characteristics are summarized in Fig. 1, which visually contrasts the advantages and disadvantages of each graft type.

Despite their clinical utility, traditional grafts have notable shortcomings, motivating the development of biomaterials to overcome these barriers.

## 3 Advancements in biomaterials

**Fig. 2:**
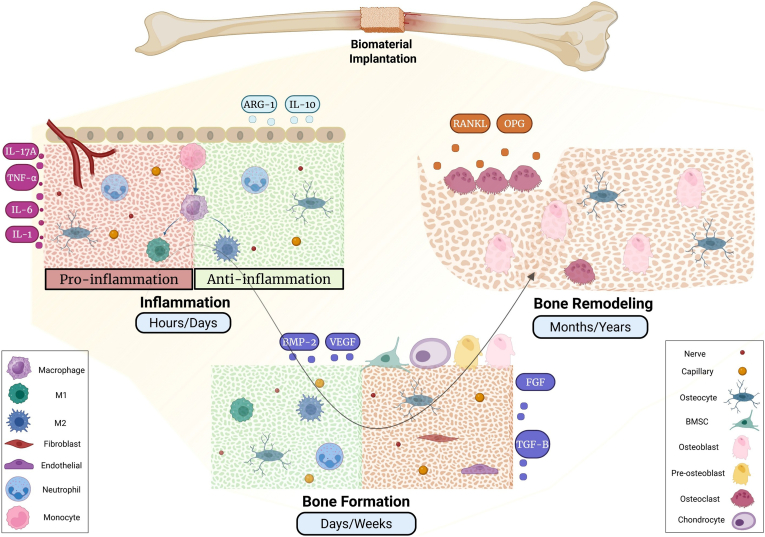
The schematic represents the healing that occurs after a biomaterial is implanted in the defect site. Inflammation (hours–days): After implantation, platelets, neutrophils and monocytes rapidly arrive and form a provisional matrix and trigger cytokine release. Neutrophils clear out the initial debris of damaged tissue while monocytes arrive later and become macrophages that help finish the job. Over time, the macrophages switch types from a pro-inflammatory one to a restorative kind and encourage other cells to help. Bone formation (days–weeks): MSCs migrate into the site and differentiate into osteoblasts, where they make osteoid that subsequently becomes mineralized woven bone. Endothelial cells make new blood vessels, and fibroblasts and osteoprogenitors coordinate matrix deposition. This stage is maintained by balanced inflammation, adequate vascularity, and active bone deposition by osteoblasts. Woven bone is resorbed by osteoclasts and by osteoblasts are replaced with organized lamellar bone. Osteocytes are embedded in the matrix and play a primary role in adapting bone structure to its mechanical environment. Over time, bone responds to mechanical cues from its surroundings and is constantly renewed and reshaped by the balanced deposition (by osteoblast cells) and resorption (by osteoclast cells) of bone, producing mature bone that is stabilized at the implant site.

Recent innovations in biomaterials have markedly improved biocompatibility, durability, and functionality. These advances have enabled faster healing, enhanced tissue regeneration, and expanded applications in implants and drug delivery. To create an advanced biomaterial suited to clinical needs, it is crucial to first comprehend the sequential stages of bone healing after biomaterial implantation. As illustrated in Fig. 2, the healing process begins with an acute inflammatory phase marked by the rapid adsorption of serum proteins onto the biomaterial's surface, which directs immune system recognition. Neutrophils are the initial immune cells to arrive, followed by macrophages that first display an M1-like pro-inflammatory phenotype before shifting to a pro-healing M2 phenotype. This early immune response is heavily influenced by biomaterial characteristics such as surface chemistry, wettability, nanoscale topography, mechanical stiffness, and degradation products. Together, these factors determine the composition of the protein corona and influence macrophage polarization. Biomaterials that reduce excessive inflammatory triggers and promote a timely switch from M1 to M2 macrophage states facilitate a smoother transition into the reparative phase [35,36].

After inflammation subsides, bone regeneration proceeds through the recruitment of mesenchymal stem cells (MSCs), their differentiation into bone-forming cells, deposition of osteoid matrix, and mineralization. These stages are supported by biomaterials with interconnected macroporosity, the release of bioactive ions like Ca^2+^, Si^4+^, and Mg^2+^, osteoinductive surface chemistries, and mechanical properties that closely resemble the native defect environment. In the final remodeling phase, the initially formed woven bone is remodeled into lamellar bone. Long-term, stable osseointegration depends on factors such as surface stability, suitable degradation or corrosion resistance, balanced osteoimmunomodulatory effects, and physiological load transfer. Biomaterial systems designed to optimize these features throughout all healing phases achieve the most reliable and functional integration results [37,38].

Building on established principles of bone healing and scaffold integration, the subsequent sections explore how various biomaterial classes employ distinct mechanical and biological strategies. Mineral-based materials are first considered, as they closely mimic the inorganic phase of bone and provide inherent osteoinductive cues, rendering them essential for load-bearing applications. The focus then shifts to natural polymers, which are characterized by low stiffness, high biocompatibility, and the capacity to promote M2 macrophage polarization, underscoring their suitability for irregular, non-load-bearing defects where immune modulation and cellular proliferation are critical. Following this, synthetic polymers are examined for their controlled degradation, tunable adhesion, and precise immunomodulatory properties, which facilitate minimally invasive delivery and enable customized biological responses in complex clinical scenarios. Finally, composite materials are discussed, as they combine the advantages of both mineral and polymer systems, achieving a balance of mechanical support and biological versatility that positions them as adaptable solutions across a broad range of bone repair contexts.

### 3.1 Mineral-based materials: promoting bone regeneration, limited by brittleness

#### 3.1.1 Calcium-based bioceramics

Bioceramics, encompassing both natural and synthetic variants, are extensively employed as bone substitute materials. Among them, hydroxyapatite (HAp) stands out due to its close compositional similarity to the mineral phase of native bone [39]. HAp supports bone regeneration and osteoconduction [[40], [41], [42]]. Safi et al. synthesized mesoporous HAp particles with high surface areas and ibuprofen encapsulation for local drug delivery [43]. HAp produced via sol-gel techniques was non-cytotoxic and induced adipose stem cell (ADSC) differentiation, supporting bone regeneration [44]. Surya et al. used fish bones to produce nano HAp (nHAp), achieving high cell viability and demonstrating its potential as a biocompatible bone substitute [45]. 3D-printed HAp scaffolds with high mechanical properties and mineral content (60 wt%) enhanced cell attachment, proliferation, and osteogenesis, showing promise for large bone defect repair [46]. Moreover, incorporating miR-26a into nHAp scaffolds promoted vascularization and mineralization, improving critical-sized defect repair in rats [47]. Bioactive metal ions, including strontium (Sr), selenium (Se), zinc (Zn), and copper (Cu), exhibit multiple biological roles and have emerged as significant competition for growth factors [48]. Se, Sr, and Zn-doped HAp incorporated into polycaprolactone (PCL)-based 3D-printed scaffolds exhibited antibacterial activity, promoted mesenchymal stem cell (MSC) proliferation, and induced osteosarcoma cell apoptosis, effectively supporting bone defect repair *in vivo* [49]. Furthermore, the bilayer was constructed by Cu, Sr, and HAp bilayer with Polylactic acid (PLA) (CSHP-) acting as a bone regeneration membrane. The release of ions enhances antibacterial responses, regulates macrophage polarization, and promotes angiogenesis. The CSHP membrane regulates the transition of macrophages to the M2 phenotype and increases vascular regeneration, thereby facilitating initial inflammation subsidence and establishing a conducive environment for bone repair [48]. Furthermore, calcium phosphate (CaP) is a bioceramic that is characterized by calcium (Ca) ions and inorganic phosphate anions, which are crucial for bone metabolism. Ca promotes osteoblast growth and differentiation, extracellular matrix mineralization, and expression of growth factors. Phosphorus induces the production of proteins involved in bone metabolism [50,51]. CaP is available in various forms, including ceramics, powders, and bone cement, with the most commonly used being beta-tricalcium phosphate (β-TCP) and HAp [[52], [53], [54], [55], [56]]. Despite their promising potential in bone repair, CaP materials are limited by their mechanical strength and brittleness, making them suitable only for non-load-bearing applications [52]. Additionally, the lack of a suitable degradation rate and appropriate cure time hampers their development. Nonetheless, different types of CaP effectively manage various bone abnormalities [57]. Wu et al. prepared 3D printed CaP ceramics to implant in a rat model for repair of calvarial abnormalities. *In vitro* cellular investigations indicated that 3D printed CaP ceramics exhibited favorable biocompatibility and facilitated the osteoblastic development of BMSCs. *In vivo* rat cranial lesion implantation demonstrated that 3D printed CaP ceramics had superior osteogenic capability compared to commercial alternatives (BAM®), reaching the performance of autografts [58]. Also, Calcium phosphate cement (CPC) is a biocompatible material used in various medical applications, particularly in bone repair and regeneration. In order to be effective in larger bone defects, CaP always needs an adjuvant [59]. A study showed the ability to enhance the osteogenic potential with decellularized extracellular matrix (dECM)–based hydrogels. It demonstrated dECM/CPC hybrid hydrogel exhibited a two-fold increase in swelling ratio and improved compressive strength. At the molecular level, osteogenic markers such as alkaline phosphatase (ALP), runt-related transcription factor-2 (Runx2), and osteocalcin (OCN) were significantly upregulated, while histological analysis revealed increased numbers of osteoblasts, osteocytes, and osteons, indicating enhanced tissue maturation and bone remodeling [60]. Similarly, fluoride-doped CPCs (FDCPCs) showed concentration-dependent effects on human dental pulp stem cells (hDPSCs), with higher fluoride levels promoting greater mineralization under osteogenic conditions. These findings support the potential of ion-modified CPCs to regulate stem cell behavior and improve osteogenic differentiation [61]. What is more, clinical evidence also highlights the value of bioactive CPC formulations. In a retrospective study involving 110 patients, BMP-2–loaded CPC (BMP-2@CPC) demonstrated superior osteoinductivity and biocompatibility compared with conventional CPC [62]. Also, Amorphous calcium phosphate (ACP) serves as an intermediary phase of HAp in bone formation and displays a distinctive structure that differentiates it from its crystalline counterpart [63]. In a study, ACP combined with hydrogel to promote bone repair in a rat model. The results of *in vivo* showed that ACP accelerated bone repair and regeneration [64].

**Fig. 3:**
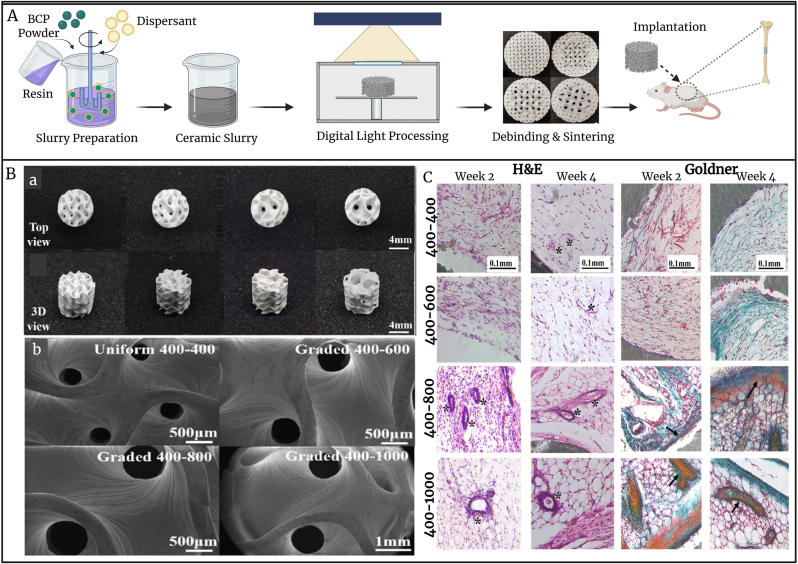
Design and fabrication, and application of 3D-printed porous scaffold gradient (PSG) for bone regeneration. (A) Schematic of scaffold fabrication in 4 different groups in pore architecture: Uniform (400-400), PSG (400–600), PSG (400–800), and PSG (400–1000). (B) SEM images of central pores showing porosity after debinding and sintering. (C) (Histological images) samples after H&E staining (black star: blood vessels) and Goldner staining (black arrow: osteoid); (Data adapted from Ref. [67]).

In addition, biphasic calcium phosphate (BCP), mixture of two CaP phases: HAp and β-TCP, a widely utilized bone substitute material, is recognized for its favorable osteogenic potential [65]. Comparative investigations assessing BCP against other bone substitute materials in dental implant applications have demonstrated that BCP supports sufficient new bone formation, surpassing xenografts in both bone regeneration and reduction of residual graft material. Although autografts and allografts remain superior in terms of regenerative capacity, BCP continues to represent a reliable and effective option for bone augmentation procedures [66]. Furthermore, recent work employed digital light processing (DLP)–based 3D printing to fabricate BCP scaffolds with different pore size gradients (PSG); Uniform (400-400), PSG (400–600), PSG (400–800), and PSG (400–1000) (Fig. 3A–B), that revealed enhanced osteogenesis *in vitro*, as well as improved bone formation and vascularization *in vivo.* As shown in Fig. 3C, two weeks after implantation, the 400–800 and 400–1000 graded scaffolds had small blood vessels, but the other two groups had little vascularization. Following four weeks of implantation, both the quantity and width of blood vessels in and surrounding the scaffolds increased in the graded 400–800 and graded 400–1000 scaffolds. Likewise, tiny blood vessels became visible in 400–400 scaffolds, and 400–600 scaffolds were graded. The results indicated that BCP scaffolds with an increased central pore size facilitated angiogenesis These scaffolds also exhibited excellent mechanical strength, highlighting their potential in clinical bone tissue engineering (BTE) [67]. Calcium sulfate bone cement (CSC) is widely used in bone regeneration and tissue engineering because of its bioactivity, ease of injection, and good mechanical properties [68,69]. Research using rabbit femur models has shown that CSC effectively promotes bone healing, supporting its use in clinical settings [69]. Recent developments have further improved CSC's therapeutic capabilities; for instance, starch-reinforced CSC containing pregelatinized hydroxypropyl distarch phosphate has been found to aid both blood clotting and new bone formation [55]. This dual action not only enhances bone repair but also efficiently manages bleeding, overcoming the drawbacks of traditional bone hemostatic materials [70]. The combination of mechanical strength, biodegradability, and biological effectiveness in these formulations makes CSC a flexible and dependable option for treating bleeding bone injuries [[68], [69], [70]].

#### 3.1.2 Silica-based bioceramics

**Fig. 4:**
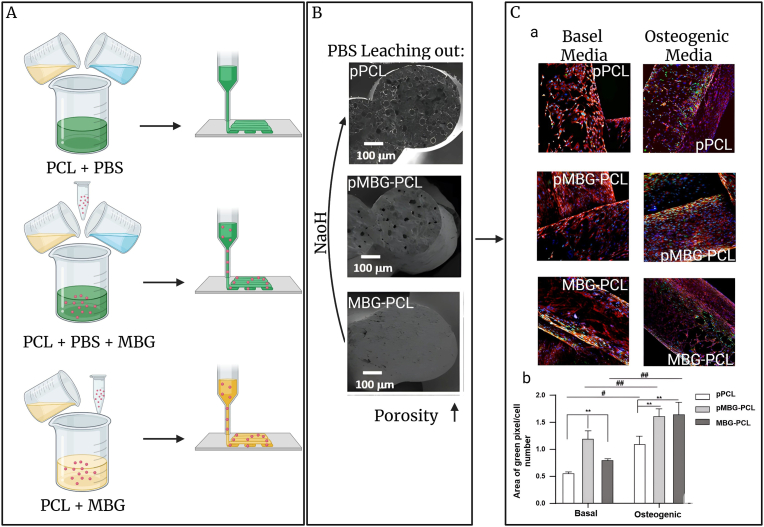
Design and characterization of 3D-printed scaffolds for bone regeneration. (A) Schematic of scaffold fabrication: pPCL, pPCL-MBG, and MBG-PCL groups. (B) SEM images showing increased porosity after NaOH salt-leaching. (C) (histological images) RUNX2 immunostaining (green), nuclei (blue), and actin (red) in basal and osteogenic media; (bar graph) Quantification of RUNX2 expression after 14 days (∗p < 0.05, ∗∗p < 0.01, ##p < 0.01 for group/media comparisons) (Data adapted from Ref. [76]).

Complementing HAp's biological affinity, Bioactive glasses (BGs), composed of Na_2_O, CaO, SiO_2_, and P_2_O_5_, further enhance osteointegration by forming an HAp-like layer in physiological fluids, thereby establishing a strong chemical bond with host bone [[71], [72], [73]]. To address the inherent fragility of BGs, engineers optimize composition, manufacturing techniques, and sintering conditions, enabling the development of porous structures that closely resemble human trabecular and cortical bone microarchitecture. Optimized sintering yields porous structures resembling trabecular bone, which promote vascularization and osteoblast migration [74,75]. Multiscale mesoporous bioactive glasses (MBGs), with their biocompatibility and bioactivity, have emerged as promising scaffolds for bone regeneration. MBGs have been incorporated into 3D-printed scaffolds to improve bone regeneration. Scaffolds made from PCL and phosphate-buffered saline (PBS)—including variations such as PCL, PCL-PBS-MBG, and PCL-MBG—exhibited microporous structures that facilitate cell and tissue colonization. Immunofluorescence analyses of RUNX2 over 14 days revealed significant upregulation of expression in scaffolds containing MBGs, supporting the role of MBGs in promoting osteogenic differentiation [76] (see Fig. 4). These findings highlight the capacity of additive manufacturing to produce patient-specific scaffolds with integrated biological functionality.

Ravanbakhsh et al. developed spherical, monodispersed MBG particles (4 nm pore size) and aminated MBGs as carriers for Alendronate to treat osteosarcoma. Aminated MBGs provided sustained, controlled alendronate release, enhancing bioactivity through HAp deposition and bioactive ion release, enabling regeneration and drug delivery [77]. Similarly, MBGs functionalized with amino (–NH_2_) groups demonstrated efficient doxorubicin encapsulation, reducing osteosarcoma cell viability without toxicity at therapeutic doses [78]. Matic et al. fabricated Sr- and Mg-doped MBG nanoparticles using sol-gel methods. These nanoparticles enhanced cell proliferation, angiogenesis, and osteogenic differentiation in human BM-MSCs and endothelial cells, highlighting their potential for bone regeneration and localized drug delivery [79]. Moreover, incorporating clove oil into MBG nanoparticles improved antibacterial efficacy against *E. coli* and *S. aureus*, while maintaining osteoblast-like cell viability [80]. MBG scaffolds enriched with osteostatin or bone marrow aspirate promoted bone formation in rabbit fracture models, achieving bone volume ratios comparable to autografts [81]. Exosome (Exo) functions as biological intercellular messengers that can influence immunological response, angiogenesis, and metabolism of bone in dental and skeletal microenvironments [82]. Wa et al. also demonstrated that Exo-treated MBGs enhanced osteoblast differentiation and angiogenesis in rat cranial defects while modulating macrophage activity [83]. Incorporating therapeutic ions such as Sr, Cu, Zn, and cobalt (Co) into MBG scaffolds has further improved bioactivity by stimulating osteogenesis and angiogenesis. For example, Cu-doped MBGs formed apatite-like layers *in vitro* and demonstrated antimicrobial properties [[84], [85], [86]]. Despite their benefits, bioceramics such as MBGs and HAp are inherently brittle, limiting their application in load-bearing scenarios. Combining bioceramics with natural or synthetic polymers enhances flexibility and toughness, creating composite scaffolds suitable for broader medical applications such as bone grafts, implants, and tissue engineering. A summary of recent research on mineral-based materials for bone applications is provided in Table 1, organized by research focus.

**Table 1:** Summary of recent research on mineral-based materials for bone applications.

Research Focus	Material/Structure	Objective	Key findings	Ref
Drug Delivery	MBG and AMBG particles	Controlled delivery of Alendronate (AL)	• Superior sustained release • Enhanced osteogenic potential	[77]
	Fe_3_O_4_/MBG nanoparticles with DOX	DOX delivery for osteosarcoma treatment	• Non-toxicity at therapeutic doses • Reduced MG63 viability	[78]
	Mesoporous nHAp with IBU	Local antibiotic delivery	• Nanoparticles (20 nm) Potential for bone regeneration	[43]
Antibacterial	MBG nanoparticles with CLV	Infection control during regeneration	• Antibacterial efficacy (*E. coli*, *S. aureus*) • Maintained osteoblast viability	[80]
	MBG with CuO nanoparticles	Evaluate bioactivity and antimicrobial properties	• Rapid bioactive response • Formation of apatite-like layers	[86]
	3D-printed Se/Sr/Zn-HAp-PCL scaffolds	Treat osteosarcoma-related bone defects	• Antibacterial properties Suppressed tumor growth	[49]
Osteogenesis	Sr/Mg-doped MBG nanoparticles	Study co-doping effects on osteogenesis	• Enhanced cell proliferation • Improved angiogenesis and osteogenic differentiation	[79]
	MBG scaffolds with osteostatin and BM aspirates	Bone formation in long bone defects	• Significant bone formation comparable to autografts	[81]
	3D porous scaffolds with HAp	Improve mechanical strength and osteogenesis	• Enhanced cell attachment and proliferation • Increased mechanical properties	[46]
	HAp with miR-26a mimics (RALA)	Deliver miRNA to enhance vascularization	• Elevated VEGF synthesis • Improved mineralization and *in vivo* bone repair	[47]
	nHAp in PLA-PHA composites	Develop biodegradable nanocomposites	• Enhanced mechanical properties	[87]
	nHAp from discarded fish bones	Low-cost method for creating biocompatible scaffolds	• Nontoxic and biocompatible • Improved cell viability	[45]
	HAp with CSC	Synthesize cement for bone repair	• Outstanding bioactivity properties • Increase mechanical properties • Enhancing bone formation	[69]
	Injectable hydroxypropyl distarch phosphate (HDP) with CSC	Develop a starch for bleeding bone treatment.	• Promoting both hemostasis and osteogenesis	[70]
	3D printed CaP ceramics	A comprehensive process for fabricating high-performance CaP ceramics to enhance bone regenerative repair capabilities	• Favorable biocompatibility and facilitated the osteoblastic development of BMSCs • Superior osteogenic capability compared to commercial alternatives (BAM®), reaching the performance of autografts	[58]
	Decellularized extracellular matrix (dECM) with CaP cement hydrogels	Develop a dECM hydrogel with CaP cement to increase bone regeneration capacity	• Enhanced osteogenic potential • Increase in swelling ratio and improved compressive strength • Accelerating tissue maturation and bone remodeling	[60]
	fluoride-doped in Cap cements (FDCPCs)	Investigate the impact of specific cements on human dental pulp stem cells (hDPSCs)	• Improved osteogenic differentiation	[61]
	BMP-2-loaded in Cap cement	To evaluate the osteoinductivity, biocompatibility, and clinical safety of BMP-2–enriched calcium phosphate cement through in-vivo testing	• Superior osteoinductivity and biocompatibility	[62]
	Injectable Amorphous CaP/PPD hydrogel scaffolds	A novel coacervate-based injectable adhesive hydrogel system for BTE	• Accelerating bone repair and regeneration	[64]
Angiogenesis	MBG microspheres + MSC-derived exosomes	Enhance bone healing and angiogenesis	• Increased osteoblast proliferation • Enhanced M2 macrophage polarization	[83]
	Cu/Sr/HAp/PLA bilayer	Suppress inflammation, promote angiogenesis, and enhance osteogenesis	• Enhanced antibacterial responses • Regulated macrophage polarization • Promoted angiogenesis • Regulated the transition of macrophages to the M2 • Facilitating initial inflammation subsidence	[48]
	3D printed biphasic calcium phosphate (BCP) scaffolds	Enhance bone healing and angiogenesis	• Enhanced osteogenesis *in vitro* • Improved bone formation and vascularization *in vivo* • Excellent mechanical strength and permeability	[67]

### 3.2 Natural-based polymers: ECM-mimicry vs. performance barriers

Natural polymers such as collagen (COL), chitosan (CS), gelatin (Gel), silk fibroin (SF), alginate (Alg), and hyaluronic acid (HA) have been extensively explored in BTE due to their biocompatibility and low immunogenicity [88]. Their structural similarity to the extracellular matrix (ECM) supports cell adhesion and proliferation and they are frequently modified chemically with cell-adhesive peptides or proteins and/or infused with osteogenic or angiogenic agents. However, poor mechanical strength, unpredictable degradation rates, and batch-to-batch variability remain major challenges [[89], [90], [91], [92], [93], [94]]. In addition to traditional biomaterials, other categories of protein-based and polysaccharide-based natural polymers have garnered considerable interest in biomedical and sustainable materials applications. Protein-based polymers sourced from plant, animal, or microbial proteins demonstrate adjustable mechanical properties, biodegradability, and may be fabricated into films, hydrogels, and scaffolds; recent developments in crosslinking and fabrication techniques have significantly improved their mechanical and physical properties [95]. Polysaccharide-based polymers, including chitosan, alginate, hyaluronic acid, and starch, are esteemed for their renewability, film-forming capacity, bioadhesiveness, and advantageous rheological properties. Contemporary techniques, such as electrospinning and chemical modification, enhance their stability and functional efficacy for across various applications [[96], [97], [98]].

#### 3.2.1 Protein-based polymers

##### 3.2.1.1 Collagen

COL, the most abundant ECM protein, accounts for more than 90 % of type I–III collagen in the vertebrate extracellular matrix [88]. Type I COL, dominant in bone and tendon tissue, supports osteoblast adhesion and growth [[89], [90], [91]]. However, COL exhibits low mechanical strength and limited osteoinductivity, necessitating functionalization or combination with other biomaterials to improve its performance [93,99]. Medium-crosslinked recombinant COL peptides derived from human COL type I show significant osteogenic capacity in animal studies, comparable in efficacy to autologous bone grafts. These peptides also recruit osteogenic cells effectively [100]. Similarly, COL scaffolds co-cultured with BM-MSCs demonstrate improved osteogenesis, antibacterial efficacy, and bone defect healing under inflammatory conditions. These scaffolds promote M2 macrophage polarization, fostering an anti-inflammatory and osteogenic environment [101]. Hydrogels synthesized from COL peptides, including those derived from Asian carp scales, exhibit strong osteogenic behavior *in vitro* and *in vivo*. However, limitations such as insufficient mechanical strength and rapid enzymatic degradation remain challenges [102,103]. Incorporation of bioceramics enhances the mechanical and osteoconductive properties of COL-based scaffolds. HAp/COL composites, antibiotic-loaded, function as drug delivery systems for osteomyelitis while retaining elasticity [102,104]. Modified COL scaffolds with BG nanoparticles promote tissue regeneration and integration, as well as cellular and BM-MSC marker expression [[104], [105], [106]]. Incorporating bioactive ions such as Cu, Co, Zn, Sr, and magnesium (Mg) further improves mechanical properties, angiogenesis, and osteogenic activity [74,107,108]. Sr-enriched COL/BG nanocomposites, for instance, enhance OCN synthesis and accelerate bone regeneration in rabbit models [109].

Despite these advances, significant challenges limit the use of COL as a biomaterial in BTE. Its poor mechanical strength makes it unsuitable for load-bearing applications, and controlling its degradation rate is difficult, often resulting in disruption of the healing process [102,[110], [111], [112]]. Additionally, COL sourced from animals carries risks of zoonotic diseases, such as transmissible spongiform encephalopathy, posing safety concerns [113,114]. Functionalization of COL is frequently required to provide the specific biochemical cues necessary for BTE, adding complexity to its use [[115], [116], [117]].

3D printing applications further highlight COL's limitations. Its low viscosity and poor structural stability hinder the creation of precise and stable 3D scaffolds. These properties lead to challenges with layer fidelity, resolution, and overall printability [118,119]. Crosslinking methods used to stabilize COL structures often produce inconsistent mechanical properties, reducing reliability in BTE [[120], [121], [122], [123]]. Addressing these limitations requires further advancements in scaffold design and material modification.

##### 3.2.1.2 Gelatin

Gel, derived from the hydrolysis and denaturation of COL from animal skin or bones, is another vital polymer in BTE [88,124]. Gel exhibits notable biocompatibility owing to the presence of Arg-Gly-Asp (RGD) in its structure, facilitating cell adhesion, dispersion, and proliferation [125]. Xu et al. investigated a Gel hydrogel system modified and cross-linked by transglutaminase (TG) and divided into distinct concentration groups. They found that the modified hydrogel promoted the infiltration, adhesion, and osteogenic differentiation of human periodontal ligament stem cells. The groups with specific TG concentrations showed elevated levels of ALP expression and mineralization, indicating favorable impacts on early osteogenic differentiation [126]. For increasing some properties, such as more osteogenesis in Gel, it can mix with different components like HA because of developing interaction with growth factors [127]. Chuang et al. synthesized Gel/HA hydrogels that crosslinked with genipin to inject into tibial bone fractures in a rabbit model. The results showed an enhancement in new bone formation, as validated by imaging and histological analysis [128]. However, the inadequate mechanical qualities prevent its direct application in bone defect therapies. Numerous research examines Gel-based scaffolds integrated with other materials to assess mechanical strength and the osteogenic development of osteoblasts. Micro and nano-additives, including BG nanoparticles, nHAp, polymer microparticles, and silica nanoparticles, can enhance mechanical stability and serve as regulated delivery systems for angiogenesis, osteogenesis, and drug agents [125,129]. Ju et al. synthesized a Gel/HAp scaffold with loading cyclic adenosine monophosphate (cAMP) and rat BM-MSCs (rBM-MSCs) for use in calvarial defects in a rat model. The results confirmed HAp/Gel scaffolds as a viable biomimetic treatment exhibiting biocompatibility and the therapeutic value of cAMP in stimulating new bone development in the skull, highlighting its potential as a growth factor for bone regeneration [125]. Gel-based sheets were produced incorporating BGs as an implant in mouse model for use in BTE after 6 weeks. The findings showed that there was great bioactivity, cytocompatibility, osteogenesis, as well as angiogenesis [130]. Also, Gel nanocomposite hydrogels were fabricated by adding 5 % BG nanoparticles for bone regeneration. The results indicated that the nanocomposite scaffolds notably increased bioactivity and mechanical properties [131]. While the integration of bioceramics can enhance osteoconduction and mechanical strength, achieving an optimal balance between porosity and strength remains a challenge for researchers [104]. Porosity was a crucial factor in assessing the bone scaffold's capability for new bone formation. Too high porosity results in diminished mechanical property of the scaffold, whereas too low porosity may hinder the proliferation of the vascularization tissue and fresh osteoblasts. Appropriate porosity and distribution of pore sizes were crucial for osteoblast adhesion, migration, regeneration, and the transport of nutrients and metabolic waste [132]. El-Bahrawy et al. developed Gel composite scaffolds featuring a porous hybrid structure for increasing mechanical strength in BTE. These Gel porous hybrid scaffolds exhibited high porosity and pore size, while their mechanical properties approximated the compressive strength of cancellous bone, indicating their potential for repairing cancellous bone damage [133]. The presence of several functional groups in Gel provides chemical modification as a compelling strategy for the development of Gel-based scaffolds. Gelatin methacryloyl (GelMA), a photocrosslinkable form of Gel, is among the most extensively researched materials [134]. In a study, an injectable scaffold was fabricated by GelMA with platelet-rich plasma (PRP) to regenerate large bone defects. The GelMA/PRP hydrogels exhibited adequate mechanical properties to facilitate the implantation of the tissue defects. The hydrogels significantly enhanced cell migration and angiogenesis in the biofunction assay. In particular, the vascularization and biomineralization capabilities of GelMA/PRP hydrogels improved and they were implanted in rats for a duration of four weeks to assess initial biocompatibility, followed by transplant in a tibial defect to simulate up to eight weeks in rats. Tibia defects addressed with GelMA/PRP hydrogels demonstrated substantial bone repair, including biomineralization, angiogenesis, and COL deposition [135]. Li et al. also isolated Exos from osteogenically pre-differentiated hBM-MSCs via ultracentrifugation and embedded in GelMA hydrogel to create a composite scaffold. The results demonstrated superior mechanical properties and biocompatibility, enabling prolonged delivery of MSC-Exos. Furthermore, osteogenic pre-differentiation substantially improved the osteogenic as well as angiogenic activities of MSC-Exos, facilitating the osteogenic metabolism of hBMSCs and the vascularization of human umbilical vein endothelial cells (HUVECs) in BTE [136].

Gel has emerged as a cost-effective and abundant material capable of encapsulating bioactive molecules like growth factors to enhance bone regeneration [137,138]. However, due to its inadequate mechanical properties, researchers have modified Gel or integrated it with various natural or synthetic polymers, bioactive ceramics, and inorganic metal/nonmetal materials to improve its structural and functional performance in BTE scaffold applications [51,139].

##### 3.2.1.3 Silk fibroin

**Fig. 5:**
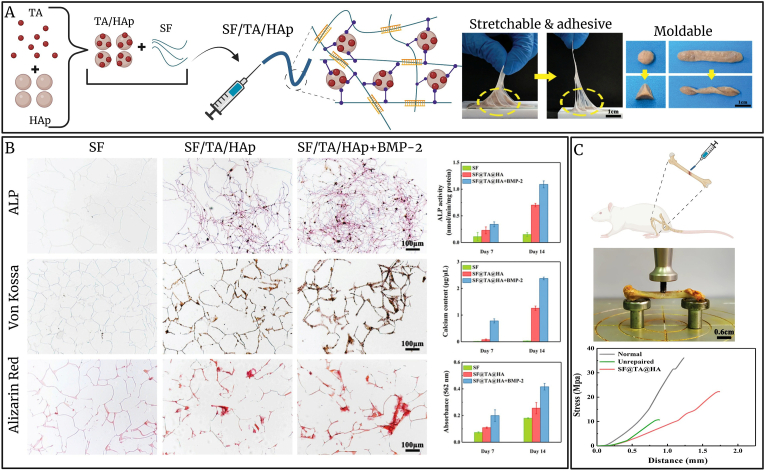
Design of an adhesive hydrogel for bone fracture repair. A) Schematic of SF/TA/HAp) synthesizing, peeling process of SF/TA/HAp on the surface of the substrate and the shapeable and moldable behaviors of SF/TA/HAp. B) *In vitro* osteogenic differentiation of rat bone MSCs seeded on 3 different groups: I) SF, II) SF/TA/HAp, and III) SF/TA/HA/BMP-2. ALP, von Kossa, and alizarin red staining were used to characterize the osteogenic differentiation of MSCs. ALP activity and contents of calcium deposition were also measured. C) *In vivo* evaluation of SF/TA/HAp fixation after 8 weeks implantation (Data adapted from: [148]).

SF demonstrates promising potential in BTE due to its superior biocompatibility, significant porosity, and strong mechanical properties. Its degradation rate aligns with the natural bone repair cycle, offering substantial benefits for bone defect materials [[140], [141], [142]]. SF is characterized by its fibrous structure. SF scaffolds with minimal porosity and reduced fiber diameter can suppress the immunological activation of macrophages and T cells [104]. Yang et al. constructed an SF-based scaffold exhibiting varying porosity and fiber thickness via electrospinning and implanted in mouse model. The results verified that the response to inflammation may be modulated by various SF topologies [143]. Furthermore, Liang et al. synthesized SF hydrogels can enhance efficient bone healing in a rabbit model with extensive, weight-bearing femoral fractures. This was accomplished by establishing a conducive microenvironment marked by decreased inflammation and enhanced COL deposition in the initial stages [144]. A Real bone comprises organic and inorganic components, whereas certain proteins, such as acidic glycosaminoglycan, function as adhesive molecules to maintain the cohesion of mineralized nanofibers [145,146]. The presence of adhesive molecules offers an energy dissipation strategy wherein calcium-mediated sacrificial bonding mitigates crack formation, hence ensuring a distinctive combination of significant biomechanical strength and toughness in biological applications [147]. Bai et al. developed mineral-organic bone adhesives inspired by bone's natural structure-function relationship, aiming for water-resistant fixation and guided regeneration. Their approach used tannic acid (TA) as a phenolic adhesive that co-assembled with SF and HAp. TA formed β-sheet-rich nanofibrils with SF, while Ca^2+^-phenolic bonds with HAp enhanced mechanical adaptability. Peeling tests showed strong adhesion, with delayed fracture and detachment at the SF/TA/HAp interface (Fig. 5A). The moldable hydrogel was biocompatible, biodegradable, and adaptable to various applications. To assess bone regeneration, rat MSCs were cultured in SF/TA/HAp. Bone Morphogenetic Protein-2 (BMP-2) was added to promote osteogenic differentiation. After 14 days, ALP activity was significantly higher in SF/TA/HAp than in SF alone, with BMP-2 + SF/TA/HAp showing the strongest ALP expression and calcium deposition (Fig. 5B). *In vivo*, SF/TA/HAp-treated rat femurs demonstrated improved fracture stabilization and compressive strength (∼22 MPa), surpassing 61.7 % of normal bone strength (Fig. 5C) [148].

Biomedicine functionality has been a primary research priority for SF. Recent literature has focused on examining SF's cell adhesion, drug-loading capability, and osteoinductivity [[149], [150], [151], [152]]. Some materials are typically utilized in composite SF for hard tissue engineering to enhance biological properties [153]. Wong et al. fabricated SF/nHAp scaffold that had degradation over 24 days to modulate the macrophage response towards an anti-inflammatory phenotype, hence promoting tissue regeneration and repair. Their findings indicated that the degradation products of the SF/nHAp scaffold on day 1 caused a pro-inflammatory response, whereas those on day 24 induced an anti-inflammatory response. Furthermore, the degradation components from the SF scaffold induced a more significant anti-inflammatory response owing to the accelerated degradation of the SF scaffold and an elevated amino acid level in the degradation mixture. Also, a deeper comprehension of macrophage polarization in relation to SF/nHAp materials can facilitate the development of strategies for designing silk-based biomaterials with adjustable degradation rates, thereby influencing macrophage responses and improving their applicability in tissue engineering [154]. In another study, inspired by GelMa hydrogel, SF was cross linked by glycidyl methacrylate (SilMa), which possesses photocrosslinkable properties. They fabricated an injectable SilMa/nHAp hydrogel to address bone defects in a rat model for 10 weeks. The *in vivo* and *in vitro* results indicated that the incorporation of 10 % nHAp enhanced biocompatibility and increased osteogenic and chondrogenic processes. Additionally, the potential of this composite hydrogel to induce macrophage M2 polarization in order to perform osteochondral repair was revealed by high quantities transcriptome sequencing [155]. As mentioned before, Mg is a crucial element for bone health. In research, SF/MgO composite scaffolds were synthesized for addressing irregular bone regeneration in rat models for 8 weeks. This scaffold enhances the proliferation, attachment, and movement of osteoblasts, as well as the osteogenic metabolism of BM-MSCs *in vitro*. *In vivo* experiments indicated that the cell-free SF/MgO scaffold significantly improves bone repair in cranial deformities [156]. To enhance oxygen delivery to tissues, Aleemardani et al. engineered a three-layer composite scaffold (hydrogel-electrospun fibers-hydrogel). This scaffold comprises two hydrogel layers infused with quercetin and an intermediate layer containing calcium peroxide and SF. The middle layer's fibers reinforce the composite structure, thereby doubling its mechanical strength. Additionally, this layer contains calcium peroxide SF, which is produced via electrospinning, which facilitates oxygen transport. Meanwhile, quercetin functions as an antioxidant, promoting stable oxygen release and enhancing cellular activity. According to the results, this composite scaffold can be presented as a secure and regulated oxygen provider, which holds potential for tissue engineering applications, especially in bone [157]. SF application in bone regeneration presents specific challenges. A significant limitation is its lack of inherent osteoinductivity, requiring the addition of bioactive molecules to stimulate bone formation. Additionally, the degradation rate of SF may not always align with the rate of new bone formation, potentially leading to scaffold failure or prolonged inflammation. To address these issues, researchers have explored combining SF with osteogenic agents like HAp to enhance bioactivity and support bone tissue regeneration [[158], [159], [160]].

#### 3.2.2 Polysaccharide-based polymers

##### 3.2.2.1 Chitosan

CS is the second most prevalent biopolymer on Earth [161]. It is widely used as a bioactive scaffold due to its biocompatibility, minimal toxicity, hydrophilicity, positively charged at a normal pH level, biodegradability, and ability to promote cell adhesion [162,163]. Augustine et al. showed that CS ascorbate enhances the hydrophilicity and water uptake capacity of membranes, significantly improving the adherence of hMSCs and human umbilical vein endothelial cells [164]. CS has free amino groups that can be protonated, enabling for the modification of CS with biological groups. The protonated amino groups facilitate electrostatic interactions with proteins, lipids, DNA, or negatively charged polymers that are synthetic [165]. Despite its numerous advantages, CS hydrogel exhibits weak mechanical characteristics. Therefore, it should be integrated with additional functional components to enhance osteogenic differentiation and tissue regeneration [166,167]. Bagheri et al. fabricated an electroactive hydrogel composed of CS/abiline oligomer and agarose, in which the aniline oligomer modulates the swelling, degradation rate, thermal characteristics, and conductivity of CS-based hydrogels. Due to its conductivity, the regulation of the electrical stimuli might influence the on-demand release of drugs, potentially enhancing cellular activity, development, and proliferation [168]. As previously mentioned, native bone matrix possesses a hierarchical structure consisting of protein COL Type ꓲ, glycosaminoglycans, proteoglycans and HAp [169]. In a study, a scaffold made of porous biological nHAp/CS (70/30) was created for bone regeneration in the periodontal region. The nHAp/CS scaffolds exhibited high porosity (78 %) with average pore dimensions of 200 μm, demonstrated osteoconduction up to 21 days, maintained structural stability for 28 days with a low degradation rate, and showed appropriate mechanical strength (storage modulus: 40–50 kPa). These characteristics made the scaffolds suitable for promoting bone growth in low-load-bearing defect locations, such as periodontal disease treatment. Compared to CS scaffolds, nHAp/CS demonstrated superior biocompatibility in both *in vivo* and *in vitro* conditions [170]. Also in another study, CS/HAp composite was combined with gentamicin drug (GA) for BTE applications. The results indicated that the CS/HAp/GA exhibited excellent biocompatibility and antibacterial properties, while promoting osteoblast cell proliferation [171]. Additionally, another study created a porous methacrylated carboxymethyl CS (CMCS) scaffold for the healing of bone defects. A photo-cross-linked methacrylated increased mechanical properties of the scaffolds. The scaffold also cultured with bone MSCs and the results show enhanced osteogenic differentiation. Recent studies have shown that the regulated release of particular amounts of metal ions, including Zn, Mg, and Ca, throughout the body is a cost-effective and efficient method for enhancing bone regeneration [172]. Mg is an essential component in the human body, with more than 50 % of Mg ions (Mg^2+^) deposited in bones, playing a crucial role in bone formation [173]. He et al. fabricated CS microcarriers deposited with Mg^2+^ -doped phase-transited lysozyme (PTL) for the purpose of accelerating bone regeneration. *In vitro* results showed that the composite microcarriers facilitated cell attachment and enhanced proliferation and osteogenic metabolism of stem cells, because of the synergistic actions of PTL and Mg^2+^. In addition, they promoted cell movement and induced macrophages that were activated by lipopolysaccharide to polarize the M2 phenotype. *In vivo* findings demonstrated that CS/PTL/Mg microcarriers expedited bone regeneration, improved angiogenesis, and decreased inflammation in the femoral ankle of rats [174]. The histological and microbiological findings demonstrated the capacity of the CS scaffold to promote cellular differentiation. The enhanced modification and antibacterial characteristics provide CS an exceptional option for functional bone implants, which still require further optimization to achieve clinically relevant mechanical performance.

##### 3.2.2.2 Alginate

Alg is a well-known substance with excellent scaffold-forming properties that can be beneficial for addressing organ loss or failure. Alg is regarded as biocompatible, non-toxic, non-immunogenic, and biodegradable [175]. Alg can immediately make a gel when divalent cationic ions, such as Ca^2+^, are added [176]. The rapid gelation characteristic of Alg renders it extensively utilized in calcium-rich solutions that simulate the bone environment [177]. Despite its affordability and ease of gelation, Alg lacks cell-binding motifs, which restricts cell attachment, and it experiences delayed degradation due to the absence of specialized enzymes for *in vivo* breakdown [127]. Consequently, Alg is utilized in conjunction with other materials to improve cell viability and functionality. Also, the incorporation of Gel into the Alg system little affects the mechanical, morphological, or swelling parameters but the mechanical properties are still poor to use in clinical applications [178]. Beheshtizadeh et al. composited sodium Alg with Gel for accelerating bone regeneration. The results demonstrated that, in contrast to the bare scaffolds, the composite scaffolds exhibited superior mechanical qualities as well as enhanced cell attachment, spreading, growth, and osteogenic differentiation [179]. Alg/Gel hydrogel scaffolds can sustain the long-term stability with its homogeneous porous surface topology. Also, the *in vitro* findings indicated that increased cell viability and great tissue forming potential in the Alg/gel hydrogel scaffolds [180]. So, adding some bioceramics such as, BG, HAp, and β-TCP nanoparticles into Alg/Gel hydrogel can increase the mechanical and biological properties. Wei et al. fabricated Alg/Gel/BG scaffolds with cells to optimize mechanical properties for bone applications. The results showed that the hydrogel was nontoxic and biocompatible [181]. Additionally, Alg/HAp hydrogel were fabricated with loading metformin (Met) for application in critical-sized fractures of the bone in rabbits after 6 weeks. The engineered hydrogel can be easily shaped and customized to fit the dimensions, form, and position of any cranial bone defects. The Alg/HAp/Met hydrogel displayed excellent cytocompatibility and osteoinductivity in both *in vitro* and *in vivo* experiments. Furthermore, computed tomography (CT) scan analysis verified superior bone filling in all treatment groups, particularly in the defects implanted with Alg/HAp/Met hydrogel [182]. In another research, Alg/Gel hydrogels were synthesized with varying concentrations of β-TCP nanoparticles that covered MC3T3-E1 preosteoblasts in the outer layer. The findings revealed that the incorporation of β-TCP nanoparticles could modify the degradation, swelling, and mechanical properties of the scaffolds. Biological analyses indicated that the cell viability of constructions with varying concentrations of β-TCP nanoparticles reached 90 % on day 7. The cell-laden structures containing 3 % (w/v) β-TCP nanoparticles had superior osteogenic and angiogenic properties [183]. As mentioned before about Sr, Manoochehri et al. constructed Alg/CS scaffolds with various concentrations of BG and Sr to enhance bone tissue regeneration. These scaffolds exhibited excellent and desirable properties for porosity, degradation, mechanical strength, and swelling profiles, biomineralization, antibacterial capacity, as well as cell growth and differentiation [184]. In summary, Alg has remarkable biocompatibility and flexible potential owing to its functional groups; yet its limits regarding strength and degradation remain research priority in this area.

##### 3.2.2.3 Hyaluronic acid

HA is extensively employed in hydrogel fabrication owing to its superior biocompatibility, excellent degradability, poor immunogenicity, anti-inflammatory as well as antioxidant properties, and may promote the migration of MSCs [127,178]. In a study it revealed that bone graft materials exhibited improved bone formation when combined with HA in a rat mandible defect, but minimal bone healing occurred in a defect treated just with HA [185]. Tavakoli et al. fabricated HA hydrogels with hMSCs to address for cell therapy and regenerative medicine. The findings indicated that the hydrogel improves long-term stability and facilitates cell growth and migration [186]. Nevertheless, HA hydrogel typically struggles to attach to bone defects in humid conditions due to insufficient tissue adhesion, potentially compromising the bonding of bone tissue [187]. Influenced by the adhesion chemistry of mussels, phenolic chemicals like TA and dopamine (DA) demonstrated rapid and reversible bonding with bone tissue, leading to their extensive application in enhancing the adherence of HA hydrogels [188,189]. However, phenolic chemicals create cross-links with polymers via unstable hydrogen bonds, leading to inadequate adhesion as well as mechanical strength [190,191]. The osteogenic properties of only phenol-functionalized HA hydrogels were inadequate for facilitating bone repair [190]. Liu et al. synthesized HA hydrogels with simvastatin (SIM) that were initially integrated into zeolitic imidazolate framework-8 (ZIF-8), followed by surface modification with HAp to produce SIM-loaded and HAp-modified ZIF-8 particles (SP). As the inorganic reinforcing agent, SP may further cross-link the dopamine-HA (dHA) and TA mixture through coordination interactions to create the hybrid sticky hydrogel (dHA/TA/SP). The adequate presence of phenolic groups provided dHA/TA/SP with superior bonding to tissues and antibacterial properties; however, the addition of SP markedly enhanced the mechanical strength and consistency of the hydrogel. *In vivo* investigations demonstrated that the dHA/TA/SP scaffold efficiently inhibited the ingrowth of fibrous structures and facilitated bone remodeling. In addition to its remarkable osteogenic effects, this hybrid hydrogel also exhibits favorable biocompatibility, injectability, self-healing capabilities, and antimicrobial properties [192]. In addition, HA/Gel/Exo composite scaffold was fabricated for BTE in a rat model for 8 weeks. The *in vitro* results indicated that the Gel/HA/Exo combination markedly improved the growth and differentiation of osteoblasts, along with the deposition and formation of bone matrix. *In vivo* investigations showed that the use of Gel/HA/Exo scaffold markedly enhanced the bone fracture healing rate and enhanced healing quality, showing excellent biocompatibility and regulated degradation properties [193]. Jiang et al. also synthesized HA/Col hydrogel containing nerve growth factor (NGF) for repairing irregular bone defects in a rat model after 8 weeks. The *in vitro* results exhibited increase in osteogenic differentiation of BMSCs. Also, *in vivo*, the hydrogel demonstrated superior bone repair efficacy compared to free NGF and blank hydrogel, exhibiting a sustained NGF-enhanced neuro-osteogenic impact over 8 weeks in a rat skull critical defect model [194]. In another study, the hydrogel was constructed by HA, HAp micron size, COL to replicate the natural bone ECM. The findings demonstrated excellent deformation and mechanical properties, as well as providing a proper immune microenvironment to regulate the M2 macrophage polarization [195]. Taken together, HA often necessitate chemical modifications or composite formulations to enhance its mechanical strength and regenerative efficacy in BTE. Table 2 summarises the recent advances in natural-based polymers for bone applications.

**Table 2:** Summary of recent advances on natural-based polymers for bone applications.

Component	Research Focus	Structure	Aim	Key findings	Ref
Collagen	Osteogenesis Antibiotic Delivery	COL/HAp Composites	Investigation of the feasibility of local antibiotic administration in the acute phase of a traumatic osteomyelitis model	• Perfect osteoconductivity and biocompatibility • Useful antibiotic delivery system for osteomyelitis	[102]
	Osteogenesis	Modified COL scaffold with BG nanoparticles and Mg/Sr/Ca	Exploration of the therapeutic effects on the repair of osteoporotic bone defect	• A non-toxic behavior to cellular proliferation • Enhancement of tissue regeneration and integration with new bone formation	[106]
	Bone Regeneration	Medium crosslinked recombinant COL mRCP particles	Determination superiority potential to regenerate bone compared to the autologous bone graft	• Significant capacity to recruit osteogenic cells • Perfect ability to close the autologous bone chips	[100]
	Osteogenesis	COL/HAp with Zn Si composite scaffold	Investigation of the effects of composite scaffold on bone regeneration and angiogenesis	• Increasing bone angiogenesis • Osteogenic microenvironment creation	[107]
	Osteogenesis	COL hydrogel with MSCs and with Sr nanocomposites	Investigation of the potential capacity of hydrogel with 2 % strontium in full-thickness bone defect regeneration in the rabbit animal model	• Enhancing bone regeneration	[109]
	Angiogenesis	COL fibrils and ZnO nanowires composite scaffold with BMMSCs	Addresses the demands of both antibacterial efficacy and osteoinductive potential	• Strong osteoinductive ability • Antibacterial effects	[101]
	Osteogenesis	COL peptide loaded Alg, Calcium carbonate	Development of a novel hydrogel system that effectively releases COL peptides.	• Increasing osteogenic differentiation in both *in vitro* and *in vivo* studies	[103]
Chitosan	Osteogenesis Cell Adhesion	CS hydrogel	Improvement of cell adhesion and proliferation	• Increasing the hydrophilicity and water uptake capacity of membranes • Improving MSCs and HUVECs adhesion on the membranes	[164]
	Osteogenesis Neural Tissue Mimicry	CS/Abiline Oligomer/Agarose hydrogels	Developing a biocompatible and electroactive hydrogel that can effectively mimic the properties of neural tissues	• Enhancing cellular activity, development and proliferation	[168]
	Osteogenesis Osteoconduction	nHAp/CS composite scaffold	Exploring the scaffold's osteoinductive and osteoconductive capacity to drive the osteogenic differentiation *in vitro*	• Great biocompatibility • Showing osteoconduction after 21 days • Maintenance stability of structure after 28 days	[170]
	Osteogenesis Antibacterial	CS/HAp/GA composite	Development of a composite material that effectively combines HAp derived from mussel shells with CS and gentamicin	• Excellent biocompatible and antibacterial properties • Increasing the osteoblast cell proliferation	[171]
	Osteogenesis	CMCS/SF/Icariin composite scaffold	A multifunctional composite scaffold that addresses the challenges associated with bone defects	• Enhancement of osteogenic differentiation	[196]
	Osteogenesis Angiogenesis Immunomodulation	CS/Mg/phase-transited lysozyme composite microcarriers	CS microcarriers that are enhanced with Mg^2+^ doped PTL for their potential application in bone regeneration	• Increasing cell adhesion, proliferation and osteogenic metabolism of stem cells • Developing bone regeneration, angiogenesis, and decreasing inflammation in the rat model	[174]
Gelatin	Osteogenesis	TG modified Gel hydrogels	Exploration of the appropriate concentration ratio of biofriendly transglutaminase-modified Gel hydrogels to promote periodontal alveolar bone regeneration	• Promotion osteogenic differentiation	[126]
	Osteogenesis	Gel/HA hydrogels	Novel biofunctionalized hydrogel system for enhancing bone repair and regeneration	• Enhancement in new bone formation	[128]
	Osteogenesis	Gel/HAp with cyclic adenosine monophosphate scaffold	Investigate the potential of cAMP in enhancing bone regeneration	• Promoting new bone formation of a skull	[125]
	Osteogenesis Angiogenesis	Gel/BG base sheets	Novel biopaper incorporating BGs adapted to vascularized bone biofabrication strategies	• Excellent cytocompatibility, • Osteogenesis and bioactivity properties	[130]
	Osteogenesis Mechanical Support	Gel/BG nanocomposite hydrogels	New simple sol-gel processing method for the synthesis of BG, and then fabricate polymeric hydrogel nanocomposite scaffolds	• Increasing bioactivity and mechanical properties	[131]
	Osteogenesis Mechanical Support	Gel/HAp/PVA/Alg porous composite scaffolds	3D multicomponent hybrid porous scaffolds that can exhibit bone-like flexible spongy structures to dissipate energy in the porous matrix structure	• Increase in the mechanical strength	[133]
	Osteogenesis Angiogenesis	GelMA/PRP hydrogels	In situ injectable hydrogels using PRP-loaded and employ them for the regeneration of a large-sized bone defect	• Increasing bone regeneration, angiogenesis, • Biomineralization, and COL deposition	[135]
	Osteogenesis Angiogenesis	GelMA/EXos composite scaffold	Investigate the potential of EXos derived from osteogenically pre-differentiated hBMSCs when encapsulated in hydrogel for promoting bone regeneration	• Superior mechanical properties and biocompatibility, enabling prolonged delivery of MSC-Exos • Improving osteogenic and angiogenic activities	[136]
Silk Fibroin	Osteogenesis Immunomodulation	SF injectable scaffold	How the architecture of SF scaffolds influences the immune responses and the engraftment of BM mononuclear cells during cell therapy in bone regeneration	• Increasing anti-inflammatory reaction by different SF scaffold topography	[143]
	Osteogenesis Inflammation Control	3D printed SF hydrogel scaffold	An innovative strategy for constructing SF-based bone scaffolds that facilitate the regeneration of large-size and weight-bearing bone defects	• Decreasing inflammation and enhancing COL deposition in the initial stages	[144]
	Osteogenesis Mechanical Support	SF/HAp/TA hydrogels	Developing a novel type of bioinspired mineral-organic bone adhesive that can provide stable fixation for bone fractures while promoting accelerated bone regeneration	• Enhancement of mechanical properties • Great biocompatibility and biodegradation • Increasing the fixing efficacy of existing bone adhesives and accelerating bone regeneration during bone remodeling	[148]
	Osteogenesis Immunomodulation	SF/nHAp composite scaffold	Macrophage response to the SF/nHA scaffold in comparison	• Inducing an anti-inflammatory response after 24 days • Accelerating degradation of the SF scaffold and an elevated amino acid level in the degradation mixture • Influencing macrophage responses and improving their applicability in tissue engineering	[154]
	Osteogenesis Immunomodulation	SilMa/nHAp injectable hydrogel	A novel injectable and photocurable hydrogel composed of methacrylate-SF and nHAp for bone regeneration	• Enhancing biocompatibility and increasing osteogenic and chondrogenic processes • Inducing macrophage M2 polarization to perform osteochondral repair	[197]
	Osteogenesis Angiogenesis	SF/Mg composite scaffold	A biomaterial that addresses the challenges associated with the regeneration of critical-size bone defects, particularly those with irregular shapes	• Enhancing the proliferation, attachment, and movement of osteoblasts • Increasing the osteogenic metabolism of BMSCs *in vitro* • Significantly improving bone repair in cranial deformities *in vivo*	[156]
	Osteogenesis Oxygen Release	SF/Calcium Peroxide/quercetin hydrogel	Development of composite scaffolds for the controlled release of oxygen based on SF	• Enhancing cellular activity • Almost more than 40 mmHg of oxygen release after 13 days	[157]
Alginate	Osteogenesis Mechanical Support	Alg/GelMA/C_3_S composite scaffold	Incorporate the C_3_S ceramic powders in the Alg matrix and fabricate C_3_S-included bone scaffolds for BTE applications	• Superior mechanical qualities • Enhanced cell attachment, spreading, growth, and osteogenic differentiation	[179]
	Osteogenesis Tissue Formation	Alg/Gel hydrogel scaffold	A simple and green synthesis route with controlled use of Alg and TA for near-field electrospinning assisted 3D and 4-axis printability	• Long-term stability • Increasing cell viability and great tissue formation	[180]
	Osteogenesis Mechanical Support	Alg/Gel/BG composite scaffolds	Optimization of the mechanical and biological properties of composite hydrogels by incorporating BG nanoparticles	• Nontoxic and biocompatible • Enhancement of the mechanical properties with increasing concentration of BG.	[181]
	Osteogenesis Bone Regeneration	Alg/HAp/Met hydrogel	An injectable hydrogel composed of Alg, HAp, and Met to assess its effectiveness in promoting bone regeneration	• Excellent cytocompatibility and osteoinductivity in both *in vitro* and *in vivo* experiments. • CT scan analysis verified superior bone filling in all treatment groups, particularly in the defects addressed with Alg/HAp/Met hydrogel	[182]
	Osteogenesis Angiogenesis	3D printed Alg/GelMA/nano β-TCP/MC3T3-E1 scaffold	Feasibility of fabricating a vascularized tissue-engineered bone model	• Modifying the degradation, swelling, and mechanical properties • Increasing cell viability of constructions with varying concentrations of β-TCP nanoparticles reached 90 % on day 7 Superior osteogenic and angiogenic properties	[183]
	Osteogenesis Angiogenesis Antibacterial	Alg/CS/Bioglass/Sr hydrogel scaffold	Develop and evaluate new composite scaffolds for BTE	• Excellent and desirable properties for porosity, degradation, mechanical strength, and swelling profiles, biomineralization, antibacterial capacity, as well as cell growth and differentiation	[184]
Hyaluronic acid	Osteogenesis Cell Delivery	HA/hMSCs hydrogel	Develop an advanced bioink for 3D bioprinting that effectively supports the delivery and functionality of hMSCs	• Improving long-term stability • Facilitating cell growth and migration	[186]
	Osteogenesis Cell Adhesion Mechanical Support	dHA/TA/HAp modified hydrogel	Developing nanocomposite adhesive hydrogel with tissue adhesion and osteogenic properties to facilitate integration with host tissues and bone regeneration	• Excellent connecting to tissues and antibacterial properties • Enhancement of mechanical properties • Significant osteogenic impacts	[192]
	Osteogenesis	HA/Gel/Exos composite scaffold	hydrogel containing Gel and HA, focusing on their physical and chemical properties	• Improving the growth and differentiation of osteoblasts • Enhancing the bone fracture healing rate and increasing healing quality Excellent biocompatibility	[193]
	Osteogenesis	HA/COL/NGF hydrogel	A novel injectable HA/COL hydrogel that is loaded with NGF to enhance the repair of irregular bone defects	• Increase in osteogenic differentiation of BMSCs • Superior bone repair efficacy	[194]
	Osteogenesis Immunomodulation	HA/COL/microHAp hydrogel	Investigate the effectiveness of scaffolds in promoting bone regeneration and tissue repair in cranial defect models	• Excellent deformation and mechanical properties • Proper immune microenvironment to regulate the M2 macrophage polarization	[195]

Significant advances have been made in natural polymers due to their biodegradability, biocompatibility, and low toxicity, prompting ongoing research into alternative biomaterials and hybrid material combinations. Despite these advantages, natural polymers often face limitations in mechanical strength, degradation control, biological stability, and scalability, complicating their design, processing, and application for bone defect repair. Consequently, researchers are increasingly exploring synthetic biomaterials tailored to meet specific requirements for effective bone regeneration.

### 3.3 Synthetic-based polymers: balancing mechanical strength with biodegradability

Synthetic polymers possess superior mechanical properties, consistent component proportions, and favorable processing characteristics [[198], [199], [200], [201]]. They also provide unique benefits in repeatability, scalability, and precise synthesis, rendering them a viable option for scaffold development in BTE. Diverse synthetic polymers have been investigated for their efficacy in facilitating bone regeneration, each possessing distinct features that can be modified to align with the specific needs of the goal bone tissue. PLA, poly (lactic-co-glycolic acid) (PLGA),PCL, and polyethylene glycol (PEG) have been extensively utilized as scaffolds, promoting bone tissue regeneration and enabling the regulated release of therapeutic drugs [[202], [203], [204], [205]]. Biocompatibility relates to the biological properties influenced by the physical and chemical features resulting from the chemical composition of synthetic polymers [204]. Furthermore, despite their advantages, some synthetic polymers can accelerate scaffold degradation and potentially cause inflammatory reactions [10,206,207].

The majority of synthetic polymers remain hydrophobic, rendering them less appropriate for therapeutic applications related to tissue regeneration [204]. For instance, while PLA is hydrophobic, PLGA copolymers are hydrophilic due to the presence of glycolic acid on their structure [208]. Incorporating PEG can further enhance hydrophilicity, improve cell adhesion, and reduce inflammatory responses, thereby increasing the biological functionality of the scaffold [209]. During the hydrolytic degradation of PLA, ester bonds within molecular chains are broken down by hydrogen ions, forming alcohol and carboxylic acids. The resulting carboxylic acids create a localized acidic microenvironment that adversely affects cell growth and bone healing, potentially triggering an inflammatory response. To enhance the biological functionality of these materials and promote cell attachment and growth, it is essential to incorporate growth factors, cells, or other substances [202,210,211].

#### 3.3.1 Polylactic acid

**Fig. 6:**
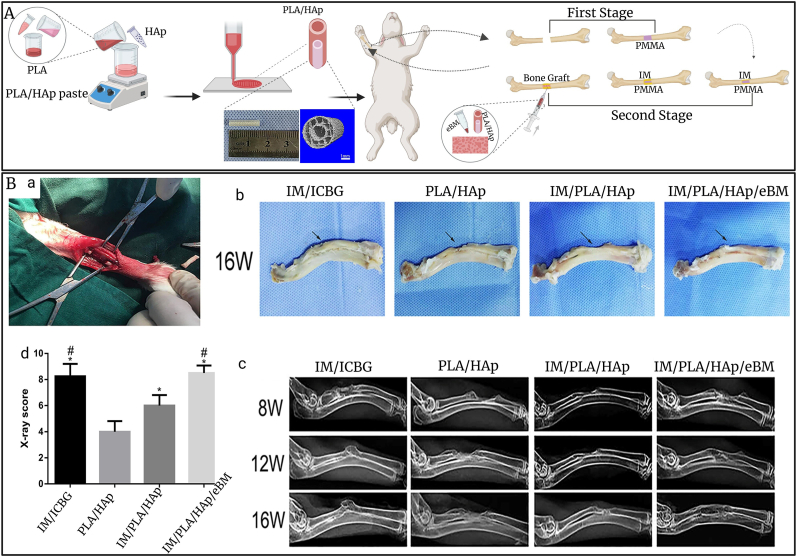
Design of 3D composite on repairing long bone defects *in vivo*. A) The schematic of healing process of induced membrane (IM) in conjunction of 3D PLA/HAp and enhanced bone marrow (eBM) for the treatment of rabbit radius long bone defect. B) a) surgical process, b) the gross specimen can observed in 4 different groups: I) IM/iliac crest bone graft (ICBG) IM/ICBG, II) PLA-HAp, III) IM/PLA-HAp, and IV) IM/PLA-HAp/eBM at 16 weeks, c) radiographs of radial long bone defect in all groups at 8, 12, and 16 weeks, d) mean scores of X-ray of all groups taken at 16 weeks post-operatively using Lane-Sandhu X-ray scores (Data adapted from: [227]).

In recent years, PLA has been increasingly used in tissue regeneration scaffolds due to its biocompatibility, biodegradability, and mechanical properties, especially when created using the electrospinning technique [212]. Some studies indicated that PLA scaffolds facilitate adhesion, growth and differentiation of osteoblast cells [213,214]. Furthermore, to enhance osteoinductive potential, synthetic polymers are altered by modifications in surface topography and chemical composition [[215], [216], [217], [218]]. Surface topography is essential in cell-scaffold connections, facilitating interaction guidance for cell growing, proliferation, as well as differentiation through cytoskeletal structure and cellular dynamics [219,220]. Thus, in research by Wang et al. fabricated PLA scaffolds featuring chemically developed nano-topographical patterns. The impact of integrated patterns in bone healing was assessed utilizing an *in vivo* rat femur critical-sized fracture model. The Micro-CT evaluation and histological examination indicated that the scaffold equipped with integrated patterns enhanced osteogenesis more effectively than the scaffolds without patterns and those solely featuring nano-topography [221]. The integration of PLA with bioceramics like BG, β-TCP, and HAp improves the inherent characteristics of PLA, providing these composites suitable for diverse biological applications. Bioceramics possess strong compressive strength, that, when integrated with PLA, markedly improves the total mechanical strength of the combination [222,223]. In a study, they aimed to increase the biological activity of PLA by adding BG to promote the new bone formation. Thus, the 3D printing filling structure was synthesized containing PLA/BG for bone regeneration. The results indicated that the BG degradation in the structure enhanced osteogenic metabolism, stimulated M2 macrophage polarization, and supported the local inflammatory reaction. The implantation of the filling structure substantially increased regeneration in the femoral bone fracture region in the rabbit models [224]. Wang et al. also fabricated PLA/β-TCP scaffolds embedded with MC3T3-E1 cells for bone regeneration applications. Their findings demonstrated that increasing the amount of β-TCP in scaffolds enhanced their mechanical properties. Additionally, the PLA/β-TCP group facilitates cell proliferation, and improves the osteogenic development of the cells *in vitro* [225]. In another study, PLA/nHAp composite scaffold was synthesized for bone regeneration in a rat model after 12 weeks. The *in vitro* findings showed that the scaffolds promoted cell crawling, movement, and activated osteogenic differentiation of rBMSCs. Additionally, the *in vivo* experiments demonstrated that excellent osteogenic properties, and the process of degradation rate corresponded effectively with bone formation while confirming biosafety [226]. Furthermore, Liu et al. developed a 3D-printed PLA/HAp scaffold loaded with enhanced bone matrix (eBM) and combined it with an induced membrane (IM) to deliver growth factors for repairing large bone defects in rabbits over 16 weeks. The *in vivo* study followed a two-stage protocol (Fig. 6A): PMMA was first implanted to induce IM formation, followed by treatment with ICBG, PLA/HAp alone, PLA/HAp with IM, and PLA/HAp with IM and eBM. *In vitro* tests confirmed scaffold biocompatibility. Radiographic analysis showed progressive bone growth and integration across groups at 8, 12, and 16 weeks (Fig. 6B–c), with full recovery in the IM/PLA/HAp/eBM group, comparable to IM/ICBG. X-ray scores were significantly higher in IM/PLA/HAp/eBM and IM/ICBG groups than in PLA/HAp and IM/PLA/HAp groups (Fig. 6B–d). Micro-CT and histology further validated that PLA/HAp with eBM and IM offers a promising alternative to autografts for treating extensive bone defects [227].

#### 3.3.2 Poly (lactic-co-glycolic acid)

PLGA is also commonly used in BTE scaffolds due to its biocompatibility, biodegradability, mechanical properties and regulated degradation rate [228,229]. PLGA scaffolds have demonstrated the ability to facilitate the proliferation and development of hMSCs [230]. Thus, Barati et al. fabricated 3D PLGA macroporous scaffolds with hMSCs for tissue regeneration applications. The *in vitro* results showed enhanced stem cells proliferation and osteogenic differentiation [210]. Some research also used components like Mg, MBG, and β-TCP to increase osteogenic differentiation in PLGA scaffolds. Kim et al. synthesized the scaffold with composition of PLGA, Mg hydroxide (MH), bone ECM and bioactive polydeoxyribonucleotide (PMEP). The PMEP scaffolds exhibited excellent biological properties about cell adhesion, proliferation and differentiation into osteogenic cells *in vitro* [231]. Also, Lee et al. prepared PLGA scaffold with MH and ECM for bone regeneration in a rat model after 8 weeks. The *in vitro* and *in vivo* results demonstrated that the PLGA/MH/ECM scaffold enhances the migrating and tube-forming capacity of endothelial cells, hence accelerating bone growth and maturation [232]. In addition, Zhou et al. also engineered and constructed a composite 3D porous scaffold utilizing PLGA, β-TCP, and Mg in a rabbit model. The scaffolds demonstrated mechanical properties comparable to those of cancellous bone, as verified by the compression tests. Furthermore, the scaffolds demonstrated substantially greater mRNA levels for osteogenic genes than the control scaffolds, as validated by an *in vitro* osteogenic differentiation assay on MC3T3-E1 cells. The investigation showed that the scaffolds were transplanted that improved the cranial bone regeneration [233]. In another research, Liu et al. also constructed PLGA/MBG scaffolds to enhance bone regeneration in a rat model after 7 days following implantation. The *in vitro* results indicated an increased immune response during the initial phase. It facilitated the prompt change of macrophage phenotype from pro-inflammatory to anti-inflammatory, so establishing a more advantageous immunological milieu in the last phase, which is beneficial to enhancing vascularization and osteogenic development. Additionally, *in vivo* assessment showed that the immunological microenvironment generated by PLGA based scaffolds with 10 % MBG (P10M) promoted perfect neovascularization and abnormal bone growth [234]. Moreover, baicalein (BCL) is an active component that promotes osteoblast differentiation, facilitates angiogenesis, and reduces osteoclast differentiation [[235], [236], [237]]. Zhang et al. fabricated the multifunctional porous PLGA/BCL scaffolds that accurately conform to complex bone defect cavity. The compact upper surface of the scaffold inhibits soft tissue cell penetration, but the porous lower surface facilitates protein adsorption, cell infiltration, and cell adhesion. The scaffold's interior macro-pores with including BCL can simultaneously enhance cell differentiation, angiogenesis, and osteogenesis [237].

#### 3.3.3 Polycaprolactone

PCL is an affordable and flexible synthetic polymer approved by the U.S. FDA [202]. As an aliphatic and semi-crystalline polymer, PCL offers excellent toughness, mechanical strength, and biocompatibility [238]. PCL's proper adhesion and proliferation of cells make it advantageous for BTE. However, its slow degradation rate can sometimes hinder bone tissue healing [239]. The degradation of PCL-based scaffolds occurs through hydrolysis, eliminating the need for enzymes or catalysts [240]. Zusmanovitch et al. synthesized PCL scaffolds considering essential design criteria such as increased porosity, diverse pore sizes, rough and hydrophilic surfaces, and biofunctionalization. These scaffolds demonstrated improved cell adherence and proliferation both on the surface and within the scaffold, showing higher hydrophilicity, porosity, stiffness, and bioactivity compared to other scaffolds [241]. Also, in another study, PCL scaffold was fabricated to implant in rat models after 12 weeks for bone regeneration. The results showed that the scaffold was biocompatible and integrated with the host bone structures when it was implanted *in vivo* in a load-sharing system [242]. Because of its prolonged degradation time, PCL is used with certain bioceramics and natural polymers like MBG, β-TCP, HAp and Gel to offer better biocompatibility and biodegradability. Also, Gel helps to improve cell attachment, movement and proliferation [243]. Additionally, to address the inadequate osteogenic capacity of a polymeric scaffold, inorganic materials such as BGs may be included in the polymer matrix [244]. Hence, Fanaee et al. fabricated PCL/Gel/BG nanoparticles biocomposite scaffold coating with Fibronectin with cells and loading amoxicillin (AMX) as a model drug. Analysis showed osteogenic differentiation of cells in the scaffold. The evaluation of drug release indicated that both crosslinked and non-crosslinked fibers are suitable as drug delivery systems, based on the treatment parameters. The non-crosslinked structures exhibited a rapid release within the initial hour, but the crosslinked structures demonstrated a more consistent release, even with a reduced total release over the same duration (72 h). Scaffolds including AMX exhibited antibacterial properties against *Staphylococcus aureus*, suggesting their possible application in bone tissues vulnerable to osteomyelitis, a prevalent infection disease [245]. In a study, PCL/MBG/Sr scaffold was synthesized to enhance bone regeneration. The optimized Sr-MBGNP concentration facilitated continuous ion release, increased hydrophilicity, and bioactivity while maintaining scaffold integrity. The Sr in the scaffold greatly improved osteoblast growth and differentiation, while concurrently and efficiently suppressing osteoclast activity. All of these factors collectively created a favorable microenvironment that promotes effective bone repair [246]. In another research, PCL/MBG has been synthesized, demonstrated great biocompatibility when interacting with osteoblasts and osteoclasts *in vitro*. Implantation into cavitary defects that had been drilled in osteoporotic sheep was employed to conduct *in vivo* investigations. The scaffolds demonstrated superior bone regeneration capabilities, facilitating new bone creation in both the peripheral and interior regions, characterized by robust trabeculae, extensive vascularization, and significant numbers of osteoblasts and osteoclasts [247]. Lee et al. prepared PCL/β-TCP scaffolds to enhance bone regeneration in large bone defects in a rabbit model after 12 weeks. The *in vitro* results demonstrated that the scaffolds remarkably enhanced cell attachment. Also, *in vivo* assessment of bone regeneration at 12 weeks post-surgery indicated the greatest bone volume within the scaffold [248]. Moreover, researchers have recently developed dual-functional HAp scaffolds that enhance new bone formation while simultaneously delivering therapeutic medicines directly to the injury or illness site [249]. HAp applications are extensively utilized in the fabrication of artificial scaffolds for bone and tissue engineering due to their superior properties as a source material for synthetic bones [250]. Liu et al. prepared biodegradable PCL/HAp composites facilitate the regeneration of bone tissue. The scaffolds were used to do *in vitro* assessments which revealed great biodegradability, biocompatibility and enhancing cell proliferation. Also, the *in vivo* tests for rats and rabbits showed that the penetration of osteocytes, hence promoting the proliferation of bone cells and the regeneration of bone tissue [251]. Furthermore, PCL/HAp scaffold was synthesized to promote bone regeneration in a rat model after 30 days. The results demonstrated an increase in mechanical properties and cell viability. Additionally, *in vivo* experiments utilizing a calvarial lesion model in rats indicated that the PCL/HAp scaffold facilitated improved bone regeneration [252]. In addition to increasing bioactivity, mechanical, and osteoconductive properties, carbon nanotubes (CNTs) have expanded due to their biological qualities [253]. Surface modification approaches offer an effective method for customizing scaffold aspects and improving their interactions with adjacent tissues and cells. Multi-walled carbon nanotubes (MWCNTs) exhibit exceptional promise as surface modifiers owing to their distinctive physicochemical features, such as improved aspect ratio, electric conductivity, as well as surface functional groups [254]. Therefore, Mohammadpour et al. prepared PCL/HAp scaffolds utilizing different concentrations of MWCNT solution as coating. The *in vitro* degradation tests indicated that HAp within the PCL matrix enhanced the hydrolysis rate, leading to elevated degradation rates for the PCL/HAp scaffolds compared to pure PCL scaffolds. The MWCNT coating additionally affected the degradation behavior, including degradation rates varying according to MWCNT concentration. Cell viability improved from 84 % for pure PCL and 103 % for PCL/HAp to 109 % in the MWCNT-coated scaffolds. Furthermore, FESEM images demonstrated enhanced cell adherence on the MWCNT-coated surfaces [255].

#### 3.3.4 Polyethylene glycol (PEG)

PEG-based hydrogels have become a highly adaptable platform for BTE especially as injectable, in-situ forming scaffolds that deliver cells and/or osteogenic signals to bone defects. For example, Click-based injectable bioactive PEG hydrogels have been shown to promote rapid craniomaxillofacial bone regeneration through the controlled spatial and temporal delivery of rhBMP-2. This approach demonstrated that a thiol–end “click” hydrogel made from multifunctional PEG derivatives can be loaded with rhBMP-2 and BMSCs, then injected into a critical-size calvarial defect in rats and the defects were nearly fully repaired with strong new bone growth and increased blood vessel formation within just four weeks, highlighting the potential of PEG hydrogels as minimally invasive bone substitutes [256]. In another study, they compared “oligo-PEG” to traditional tetra-PEG hydrogels and found that the phase-separated oligo variant allowed BMP-2 to be released steadily over about 21 days, leading to complete regeneration of critical-sized mouse calvarial defects within 28 days, outperforming the conventional PEG gel [257].

More recent research has enhanced PEG hydrogels by fine-tuning their internal chemistry, such as adjusting network degradability and adding biofunctional motifs, without incorporating bulk fillers or composites. A notable example is a study on injectable hydrogels for bone regeneration with tunable degradability achieved by modifying peptide chirality. In this work, norbornene-modified 8-arm PEG macromers were crosslinked with matrix metalloproteinase (MMP)-sensitive peptide linkers whose chirality (L, D, or mixed) controlled the degradation rate. Hydrogels with L-chirality degraded faster both *in vitro* and *in vivo*, which promoted cell migration, increased expression of osteogenic markers, and enhanced bone regeneration [258]. This clearly demonstrates that a pure PEG network, when molecularly engineered, can support bone repair by synchronizing scaffold degradation with tissue formation. Additionally, researchers have started to explore PEG's capabilities beyond providing structural support by incorporating controlled release of bioactive molecules within a PEG-only matrix. For instance, a study developed a matrix metalloproteinase-responsive hydrogel with on-demand release of phosphatidylserine (PS) to promote bone regeneration through immunomodulation. This tetra-armed PEG network was crosslinked with MMP-cleavable peptide linkers and loaded with PS, enabling PS release in response to inflammation. This triggered macrophage polarization toward a pro-healing (M2) phenotype and enhanced osteogenic differentiation, resulting in new bone formation *in vivo* [259]. The study shows that PEG hydrogels can be more than passive scaffolds; even without additional filler materials, they can function as bioactive, smart delivery systems that combine injectability, adjustable mechanical properties, immunomodulation, and osteoinduction, offering a promising path for future bone repair therapies.

In conclusion, while synthetic polymers offer significant advantages in bone regeneration, such as tunable properties and cost-effectiveness, their limitations, including lack of inherent bioactivity and insufficient mechanical strength for load-bearing applications, necessitate further research and development to enhance their efficacy in clinical settings. In contrast, the majority of natural polymers are hydrophilic yet lack appropriate mechanical properties. Consequently, in the design process and to alleviate these limits, techniques like as mixing, surface modification methods, or co-polymer of natural and synthetic polymers are utilized [204]. Thus, both synthetic and natural biomaterials provide distinct advantages and disadvantages. Current studies mostly concentrate on composite materials that enhance the advantageous properties of substances while reducing their negative aspects. Table 3 summarises the recent advances in synthetic-based polymers for bone applications.

**Table 3:** Summary of recent advances in Synthetic-based polymers for bone applications.

Component	Research Focus	Structure	Aim	Key findings	Ref
Polylactic acid	Osteogenesis Bone Regeneration	3D-printed nano-topographical PLA scaffold with BG coating	3D-printed scaffolds for bone regeneration	• Increasing osteogenic differentiation in nano-topographical scaffold	[221]
	Osteogenesis Immunomodulation	3D-printed PLA/BG scaffold	An effective strategy for bone defects in weight-bearing areas	• Enhancement of osteogenic metabolism and M2 macrophage polarization • Increasing regeneration in the femoral bone fracture area	[224]
	Osteogenesis Mechanical Support	PLA/β-TCP scaffolds embedded with MC3T3-E1 cells	A scaffold that has not only high precision, texture, and roughness on the fiber surface but also has outstanding biological properties for BTE.	• Increasing the amount of -TCP in scaffolds enhanced their mechanical properties • Facilitating cell proliferation, and improving the osteogenic development of MC3T3-E1 cells	[225]
	Osteogenesis	PLA/nHAp/CS/Mg/composite scaffold	A novel tissue-engineered scaffold that effectively addresses the challenges associated with large bone defects	• Promoting cell crawling, movement, and activated osteogenic differentiation of rBMSCs. • Excellent osteogenic properties, and the process of degradation rate	[226]
	Osteogenesis	3D-printed PLA/HAp scaffold with enhanced Bone Marrow	The effectiveness of a novel approach to a 3D-printed scaffold for repairing large bone defects *in vivo*.	• Excellent differentiation in co-culturing with MSCs and e-BM-derived MSCs • Significantly Developing bone regeneration and repairing	[227]
Poly lactic-co-glycolic acid	Osteogenesis	Injectable PLGA/Gel scaffolds with hMSCs	A novel scaffold that addresses the limitations of traditional PLGA scaffolds in tissue engineering.	• Enhancement in stem cells proliferation and osteogenic differentiation	[210]
	Osteogenesis	PLGA/MH/ECM scaffold with bioactive polydeoxyribonucleotide (PDRN)	A novel biodegradable scaffold for BTE	• Perfect cell adhesion, proliferation and osteogenic metabolism	[231]
	Osteogenesis Angiogenesis	PLGA/MH/extracellular matrix composite scaffold	A multifunctional biomimetic scaffold that effectively promotes bone regeneration in osteoporotic patients	• Enhancing the migrating and tube-forming capacity of endothelial cells, hence accelerating bone growth and maturation	[232]
	Osteogenesis Immunomodulation Angiogenesis	PLGA/β-TCP/Mg composite scaffold	3D composite scaffold fabricated by low-temperature deposition manufacturing (LDM) for cranial bone regeneration.	• Higher mRNA levels compared to control scaffold • Improving regeneration of the bone in the cranial	[233]
	Osteogenesis Immunomodulation Angiogenesis	PLGA/MBG composite scaffold	Osteoimmunomodulatory effects of hierarchical PLGA-based composite scaffolds that incorporate varying amounts of MBG	• Increasing immune response during the initial phase • Immediate change of macrophage phenotype from pro-inflammatory to anti-inflammatory • Enhancing vascularization and osteogenic development • Promoting perfect neovascularization and abnormal bone growth	[234]
	Osteogenesis Angiogenesis	PLGA/BCL composite scaffold	A novel class of personalized bone regeneration scaffolds that effectively mimic the hierarchical porous structure of the periosteum-bone complex	• Boosting protein adsorption, cell infiltration, and cell adhesion • Increasing cell differentiation, angiogenesis, and osteogenesis • Protecting and regulating the osteogenic microenvironment, leading to outstanding repair of bone defects *in vivo.*	[237]
Polycaprolactone	Osteogenesis	PCL scaffold	An improved scaffold for BTE, which is tailored to crucial scaffold requirements for a successive transplant.	• Improving cell adherence and proliferation	[241]
	Osteogenesis Biocompatibility	PCL scaffold	Biodegradable scaffold for promoting bone regeneration	• Biocompatibility and integrated with the host bone structures after implantation	[242]
	Osteogenesis Antibacterial	PCL/Gel/BG bio-composite scaffold with FN coating adding cells and AMX	A biocomposite scaffold that enhances osteogenic differentiation through the incorporation of a bio-interface.	• Suitable antibacterial properties • Consistent AMX release after 72 h • Increasing osteogenic differentiation with FN interface	[245]
	Osteogenesis Mechanical Support	3D-printed PCL/MBG/Sr scaffold	Assessment of its potential to stimulate osteoblast activity and suppress osteoclast differentiation, thereby offering a promising therapeutic avenue for the treatment of osteoporosis	• Optimal Sr-MBGNP concentration facilitated continuous ion release, increased hydrophilicity, and bioactivity Improving osteoblast growth • Promotion an effective bone repair	[246]
	Osteogenesis Angiogenesis	MBG/PCL macro-porous scaffold	Macro-porous scaffolds for bone regeneration in an osteoporotic sheep model	• Superior bone regeneration capabilities and facilitating new bone formation • Extensive vascularization • Significant numbers of osteoblasts and osteoclasts	[247]
	Osteogenesis Mechanical Support	Alg microbeads and PDA-coated 3D-Printed PCL/β-TCP scaffold	Feasible and effective clinical strategy for addressing load-bearing bone defects	• Remarkably enhanced cell attachment • The greatest bone volume within the scaffold after 12 weeks post- surgery	[248]
	Osteogenesis Biocompatibility	PCL/HAp composite scaffold	Biodegradable polymeric scaffold that can effectively repair bone defects.	• Superior biodegradability and biocompatibility • Enhancing cell proliferation • Promotion of the bone cells proliferation and bone tissue regeneration	[251]
	Osteogenesis Mechanical Support	3D-printed PCL/HAp scaffolds	Develop and evaluate functionalized scaffolds that can effectively support BTE	• An increase in mechanical properties and cell viability • Improvement bone regeneration	[252]
	Osteogenesis Mechanical Support	3D-printed PCL/HAp scaffolds with MWCNTs	The effects of surface modification on the properties of the scaffolds for applications in BTE.	• Improving cell viability to 109 % • Enhancing cell adherence	[255]
Polyethylene glycol	Osteogenesis Angiogenesis	Click-based injectable PEG hydrogel with rhBMP-2 and BMSCs	Injectable scaffold for craniomaxillofacial bone defects	• Nearly full defect repair within 4 weeks • Strong new bone formation and enhanced angiogenesis	[256]
	Osteogenesis	Phase-separated tetra-armed “oligo-PEG” hydrogel with BMP-2	Compare oligo-PEG vs conventional tetra-PEG for sustained growth factor release	• Sustained BMP-2 release over ∼21 days • Complete regeneration of critical-sized mouse calvarial defects in 28 days	[257]
	Osteogenesis	8-arm PEG hydrogel crosslinked with MMP-sensitive peptide linkers (chirality-modified)	Injectable hydrogel with tunable degradability for bone repair	• L-chirality hydrogels degraded faster • Promoted cell migration and osteogenic marker expression • Enhanced bone regeneration *in vivo*	[258]
	Osteogenesis Immunomodulation	Tetra-armed PEG hydrogel crosslinked with MMP-cleavable linkers and loaded with phosphatidylserine (PS)	Bioactive, responsive PEG scaffold for bone regeneration	• Promoted macrophage polarization to M2 phenotype • Enhanced osteogenic differentiation • Facilitated new bone formation *in vivo*	[259]

### 3.4 Composite materials: multifunctional design for enhanced regeneration

**Fig. 7:**
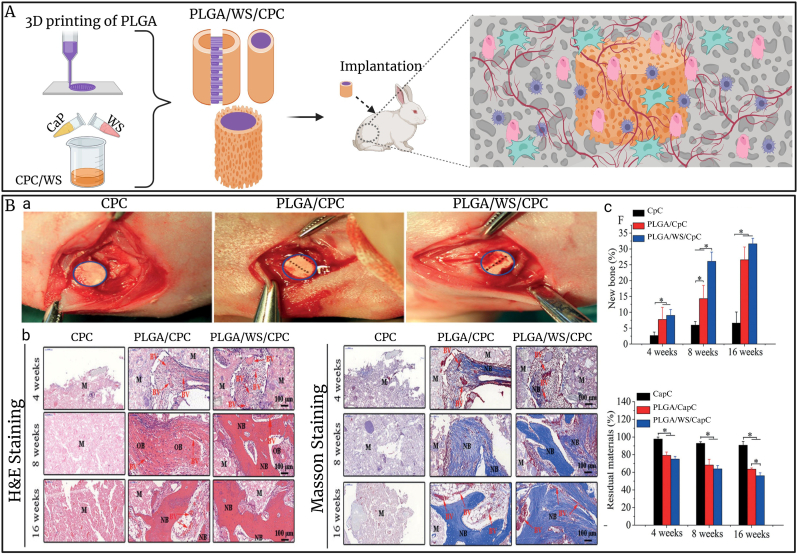
Design of 3D composite for repairing bone defects. A) Schematic diagram of CaP based composite cement with WS with 3D plotted PLGA network when implanted *in vivo* in rabbit model. B) a) Digital photographs of femoral condyle defects after implantation of three different samples, I) calcium phosphate bone cement (CPC), II) PLGA/CPC, and III) PLGA/WS/CPC, b) Histological images of H&E and Masson's trichrome staining at 4, 8, and 16 weeks after implantation, M: materials; NB: newly formed bone; BV: blood vessels; OB: osteoblast, c) quantitative results of new bone and residual materials (n = 4, ∗p < 0.05) (Data adapted from: [279]).

Composite materials are mainly divided into three categories: composites of various materials (such as bioceramics and polymers), composites of preparation technologies and materials, and composites of tissue engineering technologies and materials [93,260]. The choice of material and technique ultimately relies on the demands and qualities of the intended use [261]. Composites are essential elements in BTE, attracting significant interest in research. The combination of polymers with ceramic or bioactive compounds allows scaffolds to comply with mechanical requirements while enhancing osteoconductivity and biological recruitment [262]. In a study, Dasgupta et al. combined nHAp, β-TCP nanoparticles, and MBGs into bone scaffolds composed of Gel-CS (GCH, GCT, GCB groups). The study compared the performance of these scaffolds both *in vitro* and *in vivo*. Results indicated that GCB scaffolds exhibited a greater capacity for the proliferation and differentiation of hMSCs compared to GCH and GCT. Histological analysis of implanted bone samples at 1- and 3-months post-surgery revealed a significantly higher level of new bone production in GCB scaffolds compared to other composites [263]. In another study, the injectable CS/COL/HAp hydrogels were synthesized to create flexible, spherical micro-tissues (60–100 μm in diameter) with embedded MSCs, intended for delivery via injection. The viability and phenotype of the embedded MSCs, in addition to the shape and structure of the micro-tissues, were all analyzed. Micro-tissues were subsequently implanted into a critical-sized cranial lesion to examine the influence of cell concentration, micro-tissue prepared volume, and micro-tissue placement within the location of the defect on the amount and type of regenerated bone. The osteogenic simulation of micro-tissues indicated a remarkable advantage in accelerating bone regeneration [264]. The magnetic scaffold also was fabricated by incorporating HAp and Fe_3_O_4_ nanoparticles utilizing into CS/Col natural matrix. Furthermore, this composite has the capacity to regenerate bone tissues [265]. In another research, the Fe_3_O_4_ nanoparticles were embedded in HAp nanorods to form magnetic HAp (MagHAp). Then, GelMA/MagHAp prepared the hydrogel for implantation in a rabbit model for 12 weeks. The results demonstrated that the hydrogel was crucial in promoting cell infiltration, growth, distribution and increasing the mechanical properties [266]. In another study, Zarei et al. combined CaP to Ti6Al4V (Ti64) and PLA to create a nanocomposite scaffold for the healing of critical-size bone fractures. The scaffolds were loaded with human ADSCs (hADSCs) to assess their biocompatibility and cellular proliferation. The results indicated that the incorporation of Ti64/CaP significantly enhanced *in vitro* biocompatibility, promoting the adherence, differentiation, and growth of hADSCs [267]. In addition, CaP nanoparticles are used to improve remineralization capability and bioactivity, without adversely affecting bond strength [268]. In one study, 3D-printed scaffolds with CaP nanoparticles coating showed greater mineralization after 21 days of cell culture compared to scaffolds without CaP nanoparticles [269]. The use of CaP nanoparticles in 3D-printed scaffolds is widespread due to their biocompatibility, osseointegration capacity, and osteoconductivity [55]. Furthermore, CaP nanoparticles can be combined with hydrogel networks, resulting in bioinks with advantageous properties such as biocompatibility, mechanical properties, electrical conductivity, controlled drug release capability, and biosensing [[270], [271], [272]]. These scaffolds, made by mixing CaP nanoparticles with polymers like Gel, CS, COL, SF, and GelMA, enhance angiogenesis and osteogenesis, accelerating bone formation. The mixture can also be modified with substances like plasmid DNA, dexamethasone, and deferoxamine to improve drug delivery and antibacterial efficacy [55]. Also, ACP demonstrates an appropriate dissolution rate and capacity to promote macrophage aggregation or colonization, hence eliciting a substantial immunological response *in vivo* [273,274]. In a study, they used ACP with immunomodulatory RNA (imRNA) to mix with Col scaffolds to enhance bone regeneration in a mouse model after 6 weeks. Both *in vivo* and *in vitro* findings indicated that macrophage polarization seemed markedly responsive to the imRNA-ACP included COL scaffolds [274]. Also, metal ions can modulate various biological functions, such as immune regulation [275]. Zn ions are crucial in biological processes, including enzyme synthesizing, transmission of signals, and bone formation [276]. Furthermore, Zn ions regulate the immunomodulatory role of macrophages, promoting their polarization to the M2 profile [202,277]. Consequently, the integration of Zn ions into ACP can enhance its osteogenic and immunomodulatory properties. Wang et al. prepared PCL scaffolds by integrating osteoinductive ACP with immune-regulating ions Zn to establish a conducive immunomodulatory microenvironment in a rat model after 8 weeks. The *in vitro* experiments indicated that the PCL/ACP/Zn scaffold markedly enhanced macrophage cell adherence and subsequently modulated their polarization to the M2 phenotype, thereby promoting a pro-healing immunological environment. *In vivo* implantation observations demonstrated that immune-functionalized PCL-ACZP improved macrophage recruitment and improved new bone formation [278]. Additionally, Zheng et al. prepared an injectable hydrogel including SF/MBG/Alg scaffold in a rabbit model for 12 weeks. *In vitro* biological investigations demonstrated that the SF/MBG/Alg scaffold might enhance the osteogenic development of BM-MSCs. The SF/MBG/Alg scaffold may enhance the movement and creation of tubes of HUVECs. Analysis on the interaction between osteoblasts and macrophages demonstrated that the SF/MBG/Alg scaffold can modulate macrophage polarization from M1 to M2, hence establishing a conducive environment for promoting new bone production and angiogenesis. Moreover, *in vivo* experiment demonstrated that the SF/MBG/Alg scaffold can be readily utilized for maxillary sinus elevation, resulting in adequate new bone development [279]. Qian et al. developed a plastic composite cement (PLGA/WS/CaPC) using wollastonite (WS) and CaP bone cement (CPC) for bone defect repair in a rabbit model over 16 weeks (Fig. 7A). *In vitro*, WS promoted stromal cell adhesion, growth, and osteogenesis. Pure CPC served as a control to assess the impact of the 3D PLGA network. Histological analysis at 4, 8, and 16 weeks showed no inflammation or necrosis, with PLGA/CPC and PLGA/WS/CPC exhibiting significant CPC resorption near macropores and host bone, unlike pure CPC. At 8 and 16 weeks, both composites showed increased osteoblast activity and trabecular bone formation. H&E staining revealed abundant vasculature in PLGA-based groups by week 4 (Fig. 7B–b), attributed to macroporosity from PLGA degradation. Vessel density increased over time, with PLGA/WS/CPC outperforming PLGA/CPC. Quantitative analysis confirmed significantly higher new bone formation and biodegradation in PLGA composites compared to pure CPC at all-time points (Fig. 7B–c). These results highlight PLGA/WS/CPC's potential for enhancing angiogenesis, osteogenesis, and scaffold performance in bone defect repair [280].

Furthermore, in some research, hesperidin (HPN) represents a mono-methoxylated flavanone derived from citrus fruits, particularly oranges, and exhibits several biological effects including antioxidant, neuroprotective, and anti-inflammatory properties. HPN influences bone by inhibiting osteoclast activity. Therefore, Taymouri et al. also fabricated an injectable and biocompatible hydrogel by using HAp and HPN loading poly (3-hydroxybutyrate-co-3-hydroxyvalerate acid) nanoparticles (PHBV NPs) as a scaffold for BTE. By increasing nanoparticles to scaffolds, the mechanical properties of them were enhanced and the weight loss and swelling ratio of scaffolds decreased. Also, Lai et al. fabricated PLGA/β-TCP/Mg (PTM) scaffold in a rabbit model after 12 weeks. *In vitro* findings indicated that the PTM scaffold exhibited a well-engineered biomimetic structure and enhanced mechanical properties. Additionally, *in vivo* results displayed that the PTM scaffold possessed both osteogenic and angiogenic properties, which synergistically enhanced new bone production and improved the quality of the newly created bone [57].

The final category includes both natural and synthetic polymers, which can be combined with or without bioceramics to form a composite scaffold. Also, Pei et al. prepared an injectable scaffold including GelMA and Mg hybrid (MgH_2_) nanoparticles encapsulated in PLGA. During degradation, the scaffold releases Mg hydroxide (Mg-(OH)_2_) from MgH_2_, whereas the degradation of PLGA generates lactic and glycolic acids, which dissolve the Mg (OH)_2_ layer and produce Mg^2+^. These ions are crucial in altering macrophage morphologies, mitigating the pro-inflammatory environment, as well as regulating inflammation. They contribute to the mitigation of hyperglycemia-induced microvascular complications, hence promoting angiogenesis and vascularization, which are crucial for bone regeneration. Furthermore, hydrogen (H_2_) produced during breakdown alleviates oxidative stress by reducing reactive oxygen species (ROS). This comprehensive strategy not only diminishes reactive oxygen species and inflammation but also promotes M2 macrophage differentiation and cellular migration, resulting in enhanced angiogenesis and bone regeneration. This scaffold showed considerable potential for repair and regeneration of diabetic bone abnormalities [281]. Furthermore, in another study, the nHAp/CS/PLGA composite scaffold was constructed by loading ADSC-Exo and co-cultured with BMSCs to promote bone regeneration. The scaffold was implanted in a rabbit model for 12 weeks. The results demonstrated that ADSC-Exo can promote the osteogenic differentiation of BMSCs. The nHAp/CS/PLGA scaffolds may enhance the healing of critical-sized mandible defects in rabbits [282]. In addition, polyvinyl alcohol (PVA) is a synthetic polymer that can replicate the scaffold role of native tissue response when combined with different polymers and materials. Hence, Habibi et al. prepared PVA/CS/HA composite scaffold in a rat skull fracture model after 8 weeks. The findings indicated that cell adhesion and spreading on the scaffold, along with cell-to-cell and cell-to-scaffold interaction, enhanced with the application of HA in the scaffold matrix. Furthermore, the *in vivo* tests demonstrated that the high-dose treatment group displayed greater ossification along the defect. This was characterized by a higher quantity of active osteoblasts and ossification within the osteoid, as well as more symmetrical restoration (following 8 weeks) compared to the bare scaffold or low-dose treatment groups [283]. In another study, the core-shell scaffold was prepared by PLA/CS as a core in the PLA/CS/Gel hydrogel scaffold that was seeded by BhMSCs. The scaffold had biocompatibility, mechanical properties and excellent cell osteogenic differentiation [284]. The aim of creating a composite is to enhance the benefits of the material in various applications. So, black phosphorus (BP), a two-dimensional (2D) nanomaterial, has recently attracted interest in bone regeneration scaffolds owing to its unique properties such as biocompatibility, bioactivity, and osteogenetic properties [285,286]. Also, Polydopamine (PDA) coating is a surface modification method inspired by the adhesive proteins of mussels, allowing them to attach to wet surfaces. PDA creates a thin, conformal layer on nearly any substrate, rendering it a versatile and extensively applications due to increasing hydrophilic properties of surface and superior biocompatibility [287]. Also, deferoxamine (DFO), an FDA-approved iron-chelating agent, is used because of its prompting effects on vascularization and differentiation [288,289]. Thus, Wu et al. fabricated a novel hybrid hydrogel system composed of GelMA and sodium methacrylic acid Alg (Alg-MA) (GA), which was further integrated with a BP-based nanocomposite (BPPD), covered with PDA and infused with DFO that implanted in a rat model. The *in vitro* assessment and *in vivo* subcutaneous implanting studies revealed that the GA/BPPDM therapeutic structure exhibited exceptional biocompatibility, pro-osteogenic and pro-angiogenic properties, as well as remarkable ROS-scavenging abilities and immunomodulatory activities. The *in vivo* tests further validated that the GA/BPPDM could enhance many regenerative methods during the healing of a critical-sized bone fracture in rats, such M2 macrophage polarization, osteogenesis, neovascularization, and tissue remodeling [289]. In another study, the CS/HAp hydrogel was coated by PDA/nHAp as a hybrid scaffold in a rabbit model after 12 weeks. The CS/HAp/PDA scaffold has a structured porous design to facilitate quick cell infiltration and effective immunomodulation, hence enhancing stem cell attraction, maintenance, and final osteogenic differentiation. Transcriptomic analysis, along with *in vitro* and *in vivo* validation, indicates that the vital colony-stimulating factor-1 (CSF-1) is activated by this scaffold and successfully binds to the (CSF-1) receptor (CSF-1R) on the macrophage surface, thereby activating the M2 phenotype and facilitating significant endogenous bone regeneration [290]. Moreover, the hydrogel was synthesized by GelMA, with BMP-2 and CaP oligomers in a rabbit model. The graft demonstrated superior osteogenic and angiogenic properties *in vitro*, facilitating revascularization and reconstructing new bone with its original form *in vivo* [291]. In another study, Radwan et al. synthesized CS/CaP composites with added moxifloxacin hydrochloride. Their findings indicated that the composites achieved complete drug release within three days, stimulated osteoblast growth and specialization, and reduced bacterial presence, inflammation, and fibrosis in bone tissue samples from an animal model with osteomyelitis [163]. Table 4 provides an overview of the latest developments in composite materials for use in bone applications.

**Table 4:** Summary of the recent advances on composite materials for bone applications.

Research Focus	Structure	Aim	Key findings	Ref
Osteogenesis Bone Regeneration	Gel/CS/nHAp composite scaffolds	Developing composite scaffolds to compare bioactivities *in vitro* and *in vivo* in BTE.	• Higher ability for proliferation and differentiation of hMSCs in Gel/CS/BG scaffold • Greater level of new bone formation in Gel/CS/BG after 1- and 3-months surgery	[263]
Osteogenesis Bone Regeneration	CS/COL/HAp hydrogels with MSCs	Creation of microtissues for effective delivery to fill cavital defects in bone tissue.	• Significantly accelerating bone regeneration	[264]
Osteogenesis Bone Regeneration	CS/COL with HAp and Fe_3_O_4_ nanoparticles composite scaffold	Development and evaluation of a novel magnetic scaffold for BTE applications.	• Higher ability to regenerate bone tissues	[265]
Osteogenesis Mechanical Support	GelMA/HAp/Fe_3_O_4_ nanoparticles composite scaffolds	A novel hydrogel that incorporates continuous biophysical and biochemical gradients to enhance the repair of full-thickness osteochondral defects.	• Increasing cell infiltration, growth, distribution • Increasing the mechanical properties	[266]
Osteogenesis Mechanical Support	Ti6Al4V/PLA/CaP nanocomposite scaffolds	A hybrid structure, that is nanostructured core-shell as fillers	• Enhancing biocompatibility, promoting the adherence, differentiation, and growth of hADSCs	[267]
Osteogenesis	Ti6Al4V scaffolds modified with CaP nanoparticles	Osteogenic potential of scaffolds modified with CaP nanoparticles	• Increasing mineralization after 21 days	[269]
Immunomodulation	Amorphous CaP/imRNA/COL scaffold	Scaffold potential in constructing biomimetic mineralization material with the immunomodulatory property	• Increasing macrophage polarization	[274]
Osteogenesis Immunomodulation	3D printing PCL/ACP/Zn scaffold	The role of Zn-doped ACP (ACP/ZN) integrated into a guided bone regeneration (GBR)	• Markedly enhanced macrophage cell adherence and subsequently modulated their polarization to the M2 phenotype • Promoting a pro-healing immunological environment • Improving macrophage recruitment and enhancing new bone formation	[278]
Osteogenesis Immunomodulation Angiogenesis	An injectable hydrogel including SF/MBG/Alg scaffold	A novel injectable hydrogel that can effectively promote bone regeneration, particularly in cases of lacunar bone deficiency	• Enhancement of the osteogenic development of MSCs • Remarkably modulate macrophage polarization	[279]
Osteogenesis Angiogenesis Mechanical Support	PLGA/WS/CaPC paste	A novel strategy for enhancing bone regeneration using a composite material	• Enhancing adhesion, growth, and osteogenic development • Facilitating rapid angiogenesis and bone formation • Excellent mechanical properties and cell compatibility	[280]
Osteogenesis Mechanical Support	HAp/HPN hydrogel with PHBV nanoparticles nanocomposite scaffold	An injectable nanocomposite hydrogel to investigate osteogenic property.	• Development of the osteoclast proliferation • Increasing mechanical properties • Increasing osteogenic capacity	[292]
Osteogenesis Immunomodulation Angiogenesis	PLGA with GelMA and MgH_2_ nanoparticles injectable scaffold	A novel injectable scaffold designed to address the challenges associated with diabetic bone defects.	• Altering macrophage morphologies and regulating inflammation and cellular migration • Promoting angiogenesis and vascularization • Increasing bone regeneration in diabetic bone defects	[281]
Osteogenesis Angiogenesis Mechanical Support	PLGA/β-TCP/Mg (PTM) composite scaffold	Effectiveness of a novel porous scaffold in promoting bone defect repair	• A well-engineered biomimetic structure and enhanced mechanical properties • Enhancement of both osteogenic and angiogenic properties	[282]
Osteogenesis	PVA/CS/HA composite scaffold	The role of HA in improving the physicochemical, morphological, mechanical, and biological properties of this hybrid composite scaffold, for regenerating bone tissue	• Increasing cell adhesion and spreading • The high-dose treatment group displayed greater ossification along the defect after 8 weeks	[283]
Osteogenesis Mechanical Support	PLA/CS/Gel hydrogel scaffold seeded with MSCs	Innovative composite scaffolds that can effectively support bone regeneration and mineralization	• Biocompatible • Increasing mechanical properties • Increasing osteogenic differentiation	[284]
Osteogenesis Immunomodulation Angiogenesis	GelMA and Alg-MA/BPPD composite scaffold	A smart responsive multifunctional scaffold for enhancing bone regeneration through improved management of the immune microenvironment,	• Exceptional biocompatibility, pro-osteogenic and pro-angiogenic properties, as well as remarkable ROS-scavenging abilities and immunomodulatory activities • Enhancement of M2 macrophage polarization, osteogenesis, neovascularization, and tissue remodeling	[289]
Osteogenesis Immunomodulation	CS/nHAp/PDA composite	A new design insight for developing highly active bone regenerative biomaterials	• Enhancing stem cell attraction, maintenance, and final osteogenic differentiation • Activating the M2 phenotype and facilitating significant endogenous bone regeneration	[290]
Osteogenesis Angiogenesis	GelMA/BMP-2/CaP oligomers composite	A novel hierarchical 3D graft designed for effective craniofacial bone restoration, focusing on both the repair of bone defects and the regeneration of natural bone architecture	• Superior osteogenic and angiogenic properties *in vitro* • Facilitating revascularization and reconstructing new bone with its original form *in vivo*	[291]
Osteogenesis Immunomodulation	CS/CaP composite scaffolds	Development and evaluation of scaffolds to effectively prevent postoperative osteomyelitis.	• Reducing bacterial presence and inflammation in osteomyelitis • Stimulation of osteoblast growth and differentiation	[163]

**Fig. 8:**
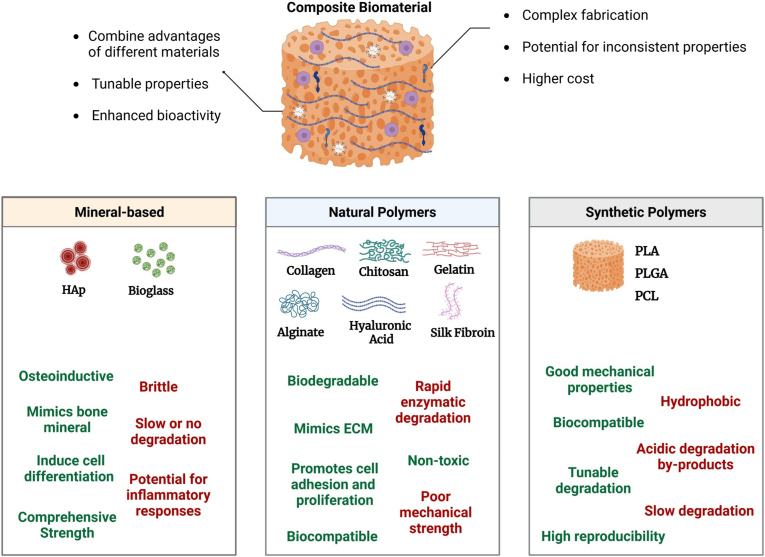
Overview of some mineral-based, natural, synthetic, and composite biomaterials used in bone repair, highlighting their key advantages (green) and limitations (red) in bone applications.

After evaluating the specific advantages and disadvantages of mineral-based, natural, and synthetic biomaterials as illustrated in Fig. 8, it is evident that no single type completely meets the varied mechanical and biological demands of bone repair. Composite biomaterials offer a promising approach by combining the benefits of different material categories into one system. By carefully integrating elements such as reinforcing polymers with mineral components or mixing natural and synthetic materials, these composites can achieve adjustable mechanical properties, increased bioactivity, and better immunomodulatory balance. This flexibility enables them to provide both structural support and biological cues, while addressing problems like the brittleness of ceramics, the low strength of natural polymers, or the harmful degradation products of synthetic materials. Although their production can be more complex and costly, composites serve as a strategic design solution that balances mechanical durability with biological performance, making them versatile scaffolds suitable for a variety of clinical bone repair applications.

## 4 Conclusion and outlook

### 4.1 Current clinical standards and limitations

Autografts, allografts, and xenografts remain the clinical standards for bone repair, but each presents inherent disadvantages. Autografts, though osteogenic and biocompatible, are limited by donor site morbidity and availability. Allografts and xenografts reduce surgical burden and expand supply but introduce risks of immune rejection, infection, and weaker mechanics. These limitations have accelerated the pursuit of engineered biomaterials as safer, more versatile alternatives.

Natural polymers such as collagen, chitosan, gelatin, silk fibroin, alginate, and hyaluronic acid provide excellent biocompatibility but are hampered by weak mechanics and variable degradation. Synthetic polymers, including PLA, PLGA, and PCL, offer tunable strength and degradation kinetics, yet lack intrinsic bioactivity and may release harmful by-products. Calcium phosphate ceramics, such as HAp and β-TCP, closely mimic bone mineral and are strongly osteoconductive, but their brittleness restricts use in load-bearing applications. These trade-offs explain why translation of engineered scaffolds into daily clinical use has been slow.

Therefore, while current grafting strategies remain effective, their limitations underscore the need for advanced biomaterials that combine biocompatibility, mechanical integrity, and biological functionality to enhance bone regeneration.

### 4.2 Scaffold design principles and translational applications

**Fig. 9:**
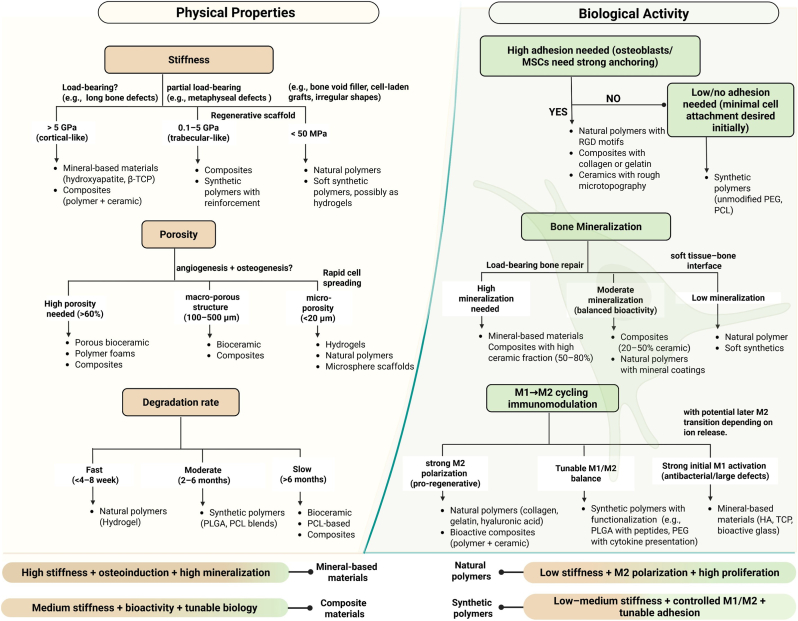
A decision tree for selecting appropriate biomaterials based on clinical requirements. The left branch focuses on physical properties such as stiffness, porosity, and degradation rate, grouping materials according to mechanical needs and structural characteristics. The right branch evaluates biological functions, including cell adhesion needs, potential for bone mineralization, and immunomodulatory effects like M1 to M2 macrophage polarization. The terminal nodes classify materials into four categories: mineral-based, composite, natural polymer, and synthetic polymer, each characterized by specific combinations of mechanical properties and dual functionality. This organized framework aids in the tailored design of bone scaffolds and regenerative constructs for specific clinical contexts.

Scaffold design for bone repair must balance mechanical and biological considerations. The decision-tree schematic (Fig. 9) synthesizes key mechanical and biological parameters that guide the selection of biomaterial classes for bone repair. It delineates how variations in stiffness, porosity architecture, degradation kinetics, immunomodulatory potential (M1→M2 macrophage polarization), cell adhesion, and mineralization capacity correspond to distinct material categories, thereby aligning scaffold design with clinical demands encountered across diverse bone defect scenarios. This integrative overview underscores the interplay between material properties and their functional roles in promoting bone regeneration, structural integrity, and localized biological responses. For example, porosity emerges as a critical design feature, particularly in scaffolds intended for vascularized bone healing [293]. High interconnected macro-porosity (100–500 μm) facilitates angiogenesis and osteogenesis [294], essential for large defect sites such as segmental long bone injuries, while micro-porosity (<20 μm) supports nutrient diffusion and rapid 3D cell network formation [295]. Specifically, microporous hydrogels created via photopolymerization-induced phase separation, with pore sizes ranging from 2 to 40 μm, enable rapid cell spreading and interconnection, allowing cells to quickly organize into a 3D network [296]. This demonstrates that controlling pore size and interconnectivity in hydrogels can efficiently guide 3D tissue-like cellular structures. Mechanical properties dictate scaffold applicability: porous mineral-based ceramics are suitable for load-bearing applications such as tibial or femoral reconstructions, providing high mechanical strength (>5 GPa) and osteoinductive signaling. Macroporous biomaterials with moderate stiffness (0.1–5 GPa) are suitable for areas that bear partial loads, such as metaphyseal defects. In contrast, soft microporous biomaterials like hydrogels, which have stiffness below 50 MPa, promote quick formation of 3D networks and rapid bone growth. This makes them ideal for non-load bearing uses, including filling bone voids in skeletal defects and aiding in the delivery of cells and growth factors.

Degradation rate is also equally pivotal: fast-degrading materials (<8 weeks) are suited for early-stage tissue formation in non-load-bearing regions (e.g., cranial or alveolar augmentation), where rapid matrix turnover supports cell proliferation and immune modulation. In contrast, slow-degrading scaffolds (>6 months) are preferable for structural support in critical-sized defects, ensuring mechanical stability during prolonged healing phases.

Biological parameters such as cell adhesion, mineralization, and immunomodulatory behavior further guide scaffold design. Materials with high cell adhesion, such as natural polymers with RGD motifs or bioactive composites, enhance osteoblast and MSC attachment, whereas unmodified synthetic polymers like PEG or PCL offer lower adhesion surfaces. Mineral-based materials and composites with high ceramic content facilitate robust mineral deposition for load-bearing repair, while soft polymers suit low-mineralization zones like soft tissue–bone interfaces. Immunomodulatory behavior is also critical: natural polymers and bioactive composites favor M2 polarization, synthetic polymers can be functionalized to tune M1/M2 balance, and mineral-based materials initially induce M1 activation to stimulate early inflammatory remodeling [297,298].

Composite materials with moderate stiffness and tunable bioactivity are suitable for spinal fusion or metaphyseal defects, where balanced mechanical support and cellular interactions are required [299]. Natural polymers and hydrogels, characterized by low stiffness and strong M2 polarization, are ideal for irregular, injectable formats in minimally invasive procedures [300]. Synthetic materials, offering adjustable stiffness and cell adhesion, are particularly valuable in osteoporotic vertebral compression fractures, where controlled degradation and localized immunomodulation enhance therapeutic outcomes [301]. These material-specific trends demonstrate how scaffold design can be tailored to meet the biomechanical and biological demands of distinct bone repair scenarios.

The principles of scaffold design are reflected in current clinical products, highlighting how engineered biomaterials translate into practical bone repair solutions. Mineral-based biomaterials such as Biolox® and Straumann PURE™ demonstrate exceptional mechanical strength and long-term stability, making them adopted for load bearing joint replacements and dental implants, respectively. Beyond bioceramics, composite systems—such as RegenOss® and FIBERGRAFT™ AERIDYAN™ Matrix, combine hydrogels, synthetic polymers, and bioceramic components to create tunable scaffolds capable of delivering growth factors, supporting early inflammation, and guiding immunomodulatory responses. These commercial products exemplify how integrating mechanical performance with biological functionality can meet diverse clinical demands, from orthopedic trauma to craniofacial reconstruction. Table 5 provides a comparative overview of commercially available biomaterials, detailing their components, manufacturers, and primary clinical applications, underscoring the breadth of material strategies currently in practice.

**Table 5:** List of some commercial clinical products available in the market for bone repair, including their manufacturers, components, and applications.

Product	Manufacturer	Components	Application
Norian® SRS (Skeletal Repair System)	Synthes/J&J	Injectable calcium phosphate cement	Bone defect filling Non-load bearing defect
Biolox®	CeramTec	Alumina/zirconia ceramics	Orthopaedic joint replacements (hip, knee prostheses)
Straumann BoneCeramic™	Straumann	Biphasic calcium phosphate granules	Dental and maxillofacial surgery
BioOss®	Geistlich	Granuls with autogenous blood or saline solution	Dental grafting
ChronOS®	Synthes	Synthetic β-tricalcium phosphate granules	Non-load bearing
Cerament®	Bonesupport AB	Injectable calcium sulfate + hydroxyapatite	Bone Void Filler
Osteomesh	Zimmer Biomet	PCL-based	Orbital Floor Fractures
FIBERGRAFT™ AERIDYAN™ Matrix	DePuy Synthes	Boron-based bioactive glass + collagen + essential ions (Mg, Zn, Cu, K)	Non-load bearing Bone void filling
OsteoBiol®	Tecnoss	Collagen-preserving processing of porcine-derived bone	Regenerate bone defects in dental surgery
Novabone IRM™	NovaBone Products LLC	premixed composite of bioactive calcium-phospho-silicate particulate and a synthetic, absorbable binder	Bone void filler
Straumann PURE™ Ceramic Implant	Straumann	Zirconia ceramic	Dental implant (esthetic, metal-free cases)
INFUSE® Bone Graft	Medtronic	Collagen sponge carrier + BMP-2 growth factor	Dental implant placement
MagnetOs®	Kuros Biosciences	Mixture of hydroxyapatite (HA) and β tricalcium phosphate (β TCP) (NeedleGrip™ technology)	Bone void filling
RegenOss®	Finceramica/JRI Orthopaedics	Collagen + hydroxyapatite composite	Filling bony voids or gaps of the skeletal system

### 4.3 Emerging strategies and future directions

Despite the current advances, key challenges remain, including achieving optimal cellular interactions, vascularization, and integration with host tissue. Next-generation scaffolds are moving toward dynamic, biologically interactive systems that coordinate osteogenesis, angiogenesis, immunomodulation, and even neural regulation [303]. Beyond simply guiding macrophage polarization, these “intelligent” scaffolds aim to systematically modulate a broader spectrum of immune cells, creating a microenvironment that actively promotes regeneration and reduces chronic inflammation [304]. Approaches such as use of neurotrophic factors, Mg^2+^/Zn^2+^-doped ceramics, exosome-functionalized scaffolds, and advanced fabrication techniques like electrospinning, bioreactors, and 3D/4D printing allow precise control over architecture, porosity, and bioactive factor delivery, enabling patient-specific and anatomically tailored constructs [302].

Furthermore, skeletal organoid technologies, especially bone organoids created by advanced 3D bioprinting, are emerging as revolutionary research instruments that can replicate mineralization, cellular diversity, and initial vascular/neuronal patterning. These organoids provide unique platforms for biomaterial testing, regeneration modelling, and predicting patient-specific responses, thereby acting as a vital link between laboratory discovery and clinically relevant translation [302,305]. Ultimately, the success of future biomaterials will rely on biologically intelligent, clinically practical designs that respond to local cues, release bioactive factors on demand, degrade in synchrony with tissue remodeling, and are scalable and regulatory-compliant. By integrating insights from immunology, vascular biology, and neural signaling, these next-generation scaffolds can coordinate multiple regenerative pathways simultaneously, establishing a new paradigm for effective and transformative bone repair.

## CRediT authorship contribution statement

**Zahra Sabouri:** Writing – review & editing, Writing – original draft, Validation, Methodology, Investigation. **Mélanie Dequeecker:** Writing – review & editing, Writing – original draft, Validation, Investigation. **Houmam Anees:** Writing – review & editing, Writing – original draft, Investigation. **Fatemeh Rastegar Adib:** Writing – review & editing, Writing – original draft, Investigation. **Reem Jamous:** Writing – review & editing, Investigation. **Junwen Zheng:** Writing – review & editing, Investigation. **Xiaolong Lyu:** Investigation, Writing – review & editing. **Sabine Stoetzel:** Writing – review & editing, Validation. **Christian Heiss:** Writing – review & editing, Resources, Investigation, Conceptualization. **Thaqif El Khassawna:** Writing – review & editing, Validation, Supervision, Resources, Project administration, Funding acquisition. **Vahid Jahed:** Writing – review & editing, Validation, Supervision, Investigation, Conceptualization.

## Funding sources

This work was supported by the 10.13039/100021828German Academic Exchange Service (10.13039/501100001655DAAD) under the BIO-METRIC project: Biomaterials and Osteoengineering – Multidisciplinary Education for Tissue Regeneration and Innovation in Collaboration (Projekt-ID: 57799097).

## Declaration of competing interest

The authors declare that they have no known competing financial interests or personal relationships that could have appeared to influence the work reported in this paper.

## Data Availability

Data will be made available on request.
